# Probing DNA damage in Rett syndrome neurons uncovers a role for MECP2 regulation of PARP1

**DOI:** 10.1016/j.stemcr.2026.102819

**Published:** 2026-01-23

**Authors:** A. Morales, E. Korsakova, N. Mansooralavi, A. Ravikumar, G. Rivas, P. Soliman, L. Rodriguez, C. Galvan, T. McDaniel, A. Lund, B. Cooper, A. Bhaduri, W.E. Lowry

## Main text

(Stem Cell Reports *20*, 102645; October 14, 2025)

Due to an error made during preparation of the final version of the manuscript, an author, Carlos Galvan, was mistakenly omitted from the author list in the originally published article. Dr. Galvan performed the analysis of the metabolomic data; these data were very important for delineating the effect of loss of MECP2 in neuronal metabolism. The author list has now been corrected to include Carlos Galvan. The authors apologize for the omission.

