# Supplementary material for: Probing DNA damage in Rett syndrome neurons uncovers a role for MECP2 regulation of PARP1
Source: Stem Cell Reports. 2025 Sep 25;20(10):102645. doi: 10.1016/j.stemcr.2025.102645 (PMC12790717; doi:10.1016/j.stemcr.2025.102645)
Supplement: Document S1. Figures S1, S2 and Supplemental methods [file mmc1.pdf]

Stem Cell Reports, Volume *20*

Supplemental Information

Probing DNA damage in Rett syndrome neurons uncovers a role for MECP2 regulation of PARP1

A. Morales, E. Korsakova, N. Mansooralavi, A. Ravikumar, G. Rivas, P. Soliman, L. Rodriguez, T. McDaniel, A. Lund, B. Cooper, A. Bhaduri, and W.E. Lowry

Supplementary Figures

Supplemental Figure 1. Supplemental images and flow cytometry plots for mitochondrial assays in Rett syndrome neurons. (A) Mitochondrial morphology in neurons was studied using confocal microscopy and immunostaining for Tom20. Images taken in 63X. (B) Flow cytometry plots of JC-1 treated Rett neurons to understand mitochondrial permeability. Data are quantified in Fig 2I. CCCP, a proton gradient uncoupler, was used as a positive control.

Figure S1

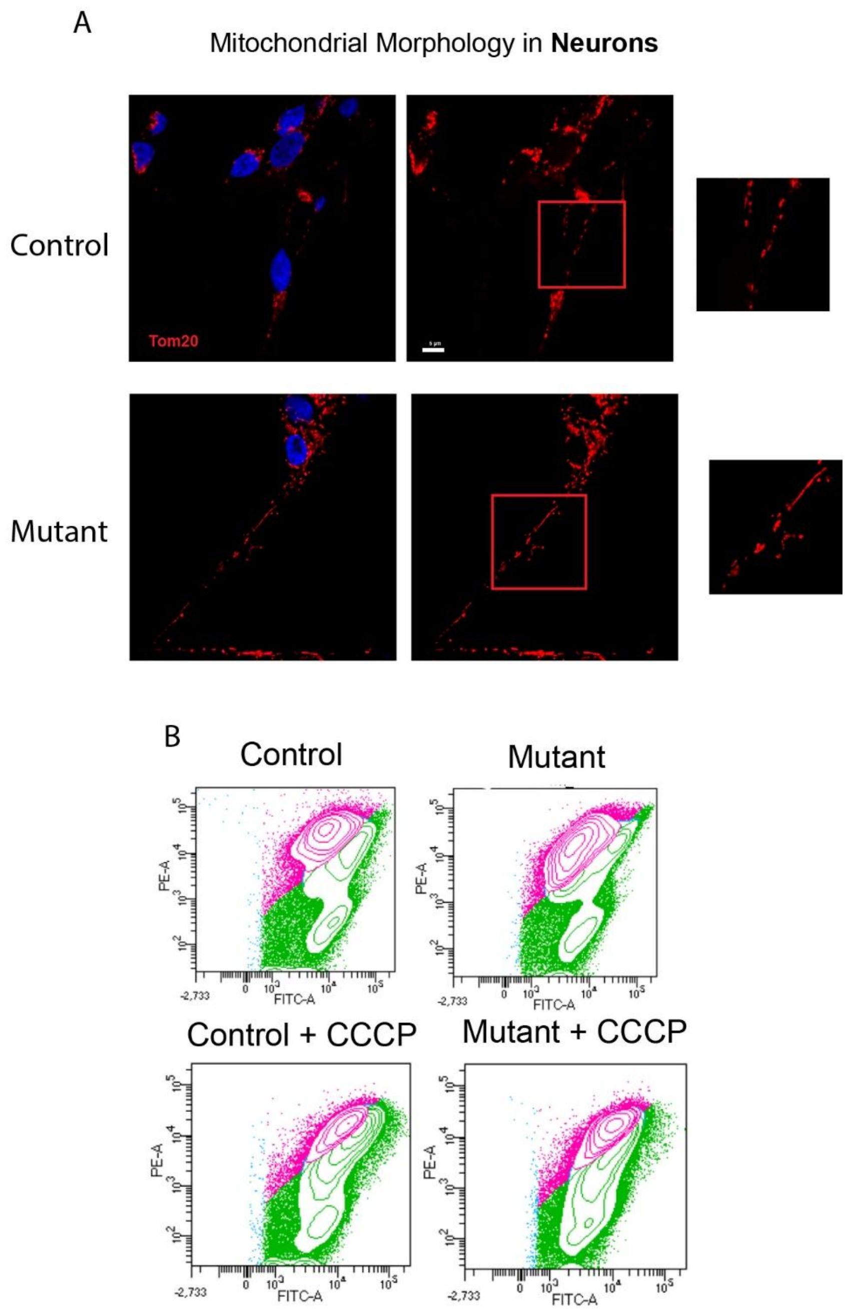

Supplemental Figure 2. Further analysis of published MECP2 protein interactomes. (A) Venn diagram of proteins identified from IP-mass spectrometry in wildtype and MECP2 mutant neurons (R15). (B) MECP2 protein interactome from OpenCell proteome database. (C) Gene ontology analysis of MECP2 interactors and their interactomes. (C) Left: Heatmap of Jaenisch proteomic database of 45 shared proteins from Figure 4E including values from Rett syndrome mutation R133C. Right: PARP1 protein quantity from Jaenisch IP-MS pull down between control and mutant neurons.

Figure S2

A

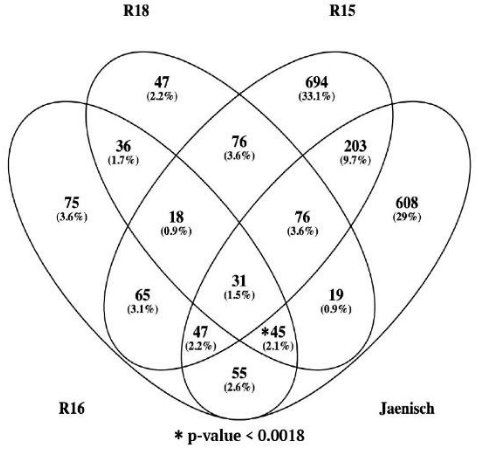

B

OpenCell MECP2 Interacations Table

Bait	Prey	P value (-log p)	Relative Enrichment	Cellular Abundance	Interaction stoichiometry	Cluster ID	Core complex
**PARP1**	**MECP2**	83.86	10.66	-2	-2.5	None	None
**H2BC11**	**MECP2**	68.6	6.55	-1.95	-2.76	None	None
**MECP2**	**MECP2**	39.64	8.33	0	0	302	336
**MECP2**	**BAZ1A**	25.43	6.14	0.41	-3.04	302	336
**KPNA4**	**MECP2**	12.32	4.15	-1.19	-2.54	None	None
**MECP2**	**CHRAC1**	9.93	4.7	0.43	-2.4	302	336
**SNX1**	**MECP2**	7.78	3.38	-0.71	-3.87	None	None
**MECP2**	**MAPK1/4**	6.85	3.19	1.66	0.01	None	None
**NUCKS1**	**MECP2**	2.56	2.06	-1.01	-2.54	None	None

Term	P value	Adjusted p value	Odds Radtio	Combined score
**Chromatin Organization**	6.32E-60	8.60495E-57	28.16207489	3838.820915
**Chromatin Remodeling**	1.53E-48	1.04273E-45	25.78368506	2838.733324
**DNA Metabolic Process**	3.82E-39	1.73507E-36	17.59838166	1556.754875
**Regulation Of DNA-templated Transcription**	7.51E-33	2.55781E-30	5.492278513	406.2573335
**DNA Repair**	2.2E-29	5.98668E-27	13.59036596	896.7947049
**DNA Damage Response**	1.18E-27	2.68858E-25	10.81257606	670.385825
**Regulation Of Transcription By RNA Polymerase II**	7.29E-23	1.41853E-20	4.217323749	214.9691231
**Positive Regulation Of DNA-templated Transcription**	1.4E-22	2.38605E-20	5.056130586	254.4211089
**Negative Regulation Of Nucleic Acid-Templated Transcription**	6.07E-20	9.18807E-18	7.61894788	337.1240452
**Positive Regulation Of Double-Strand Break Repair**	7.95E-17	5.69548E-15	20.78012685	770.3478647

C

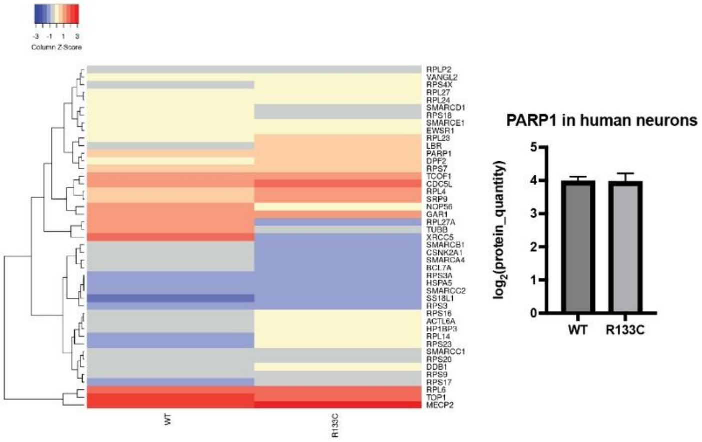

Supplemental Methods

Animals Generation of Rett syndrome rat model consisted of crosses of Mecp2ZFN/+ females (SD- Mecp2tm1sage) to wildtype (WT) S100b eGFP to produce Mecp2ZFN/y and Mecp2ZFN/+ rats. Details about this model are found in Patterson et al [32] and Morales et al (in press).

Library Preparation and Sequencing Isolated RNA (bulk RNAseq) were delivered to the UCLA Technology Center for Genomics & Bioinformatics where libraries for RNA sequencing were prepared. The samples were sequenced using NovaSeq 6000 S2 PE 2x50 with 50,000 reads per cell.

Disruption of mitochondrial function Cells were washed with PBS and treated with either DMSO, 1 uM Phenformin, 20 uM Phenformin, 0.1 uM Rotenone or 5 uM Rotenone for three days. Media was changed every day.

JC-1 Mitochondrial Permeability Assay Mitochondrial permeability was measured using ThermoFisher' s MitoProbe™ JC-1 Assay Kit (M34152) according to the manufacturer's protocol. A measure of permeability was calculated by a ratio of green and red flow cytometry events.

Reactive Oxygen Species Measurement Detection of ROS in live cells was conducted using ThermoFisher MitoSOX™ Mitochondrial Superoxide Indicators for live-cell imaging (M36007) according to manufacturer's directions.

RNA Expression Profiling RNA-seq was performed as described previously [61]. Data from experiments in figure 6 are available at Mendeley (Loss of MECP2 in human neurons - Mendeley Data)

Gene Ontology and Transcription Factor Analysis Analysis was performed using Enrichr, a gene list enrichment analysis tool. https://maayanlab.cloud/Enrichr/

Metabolic Pathway Analysis Analysis was performed using pathway analysis module of the MetaboAnalyst platform. https://www.metaboanalyst.ca/

Mitochondrial Morphology Analysis Mitochondrial morphology parameters were quantified using the MitochondrialAnalyzer plugin in ImageJ. https://github.com/AhsenChaudhry/Mitochondria-Analyzer

Confocal Colocalization Analysis The cells were analyzed by confocal microscopy using inverted confocal laser microscope Zeiss LSM880 with Airyscan at 100X. Pearson's Correlation Coefficient and was quantified using Zeiss's ZEN Blue's colocalization module.

Dendritic Branching Analysis The stained cells were then imaged at 20×, and dendritic arbors of individual cells were traced using ImageJ. The number of dendritic ends per cell were counted using the Cell Counter plugin for ImageJ. The number of dendritic ends per cell are presented as mean ends per cell ± SEM.

Western Blot Cell lysate was prepared using RIPA buffer (Pierce) supplemented with Halt Protease Inhibitor Cocktail (ThermoFisher Scientific) and Halt Phosphatase Inhibitor Cocktail (ThermoFisher Scientific). Total protein concentration was determined using BCA Protein Assay Kit (ThermoFisher Scientific) following the manufacturer's protocol. Equal protein concentrations were loaded onto the NuPAGE 4-12% Bis-Tris gel (ThermoFisher Scientific) and run at 150 Volts for 90 minutes in running buffer, containing 25 mL of 20x NuPAGE MOPS SDS Running Buffer (ThermoFisher Scientific)

and 475 mL of mili-Q water. Next, the protein was transferred onto the nitrocellulose membrane at 30 Volts for 60 minutes in transfer buffer, containing 25 mL 20x NuPAGE Transfer Buffer (ThermoFisher Scientific), 100 mL Methanol (ThermoFisher Scientific), 375 mL mili-Q water. The membrane was blocked overnight at 4°C in OneBlock Western-FL Blocking Buffer (Genesee Scientific), then incubated in the primary antibody at 4°C overnight. The following primary antibodies were used: rabbit VDAC (Cell Signaling Technology #4661, 1:1000), mouse β-actin (SCBT sc-47778, 1:500). The membrane was washed twice with 0.1% PBST and incubated in anti-rabbit or anti-mouse secondary HRP-labeled secondary antibody (ThermoFisher Scientific 31460, 31430 1:100000) for 1 hour at room temperature. The membrane was washed twice with 0.1% PBST and SuperSignal West Femto Maximum Sensitivity Substrate (ThermoFisher Scientific) was added to the membrane and subjected to film exposure.

C13 labeled glucose incorporation Cells were fed with DMEM (ThermoFisher Scientific) supplemented with 4 mM glutamine (ThermoFisher Scientific), 1 mM pyruvate (ThermoFisher Scientific), and 10 mM C13 labeled glucose (Cambridge Isotope Laboratories). 24 hours later the cells were washed twice with ammonium acetate (ThermoFisher Scientific) on ice and 80% methanol (ThermoFisher Scientific) was added to the cells. The plates were placed in -80°C for 15 minutes. Next, the cells were scraped off the plate into Eppendorf tubes, vortexed and centrifuged at 17000 g for 10 minutes at 4°C. The methanol was then evaporated using the EZ-Lite evaporator. Dried samples were analyzed using mass spectrometry with cell count normalization between samples.

Seahorse Assay Cell were plated at a density 50,000-90,000 cells per well in a XF96 microplate (Agilent) and placed in the 37oC 5% CO_2 incubator overnight. The next day, the cells were washed twice with the assay medium (Dulbecco's Modified Eagle's Medium supplemented with 10 mM glucose, 2 mM L-glutamine, 1 mM pyruvate and 5 mM HEPES, pH 7.4), and the microplate was placed in a 37°C incubator without CO_2. 30 minutes later, the plate was loaded into the Seahorse XF96 Extracellular Flux Analyzer (Agilent Technologies). The following compounds were Injected during the assay: 2 uM oligomycin, 0.75 and 1.35 uM FCCP; 2 uM rotenone and antimycin A. When the measurements were done, cells were fixed with 4% paraformaldehyde, stained with Hoechst, and cell number per well was determined using an Operetta High-Content Imaging System (PerkinElmer). Oxygen consumption rates (OCR) were normalized to cell number per well.

siRNA Gene Silencing MeCP2 protein knockdown was performed using Lipofectamine™ RNAiMAX Transfection Reagent (Invitrogen) kit. A reverse transfection method was used with a ratio of 1 μl lipofectamine:20 nM siRNA for each well of a 24-well plate. Briefly, Lipofectamine and oligos were premixed in 100 μl of OptiMEM (Gibco) for 20 min in the precoated receiving plate. Cells were then passaged with TrypLE (Gibco), resuspended in 500 μl of cell media without antibiotics, and plated on top of transfection media. Transfections were incubated overnight at 37°C after which time media was replaced with standard cell culturing media with antibiotics for the indicated lengths of time.

Cell Culture Treatments To reduce PARP activity, cells were treated with Olaparib (Selleck) at a concentration of 500 nM at indicated time points. To induce PARP activity,

β-Nicotinamide adenine dinucleotide hydrate (Sigma) was used at 10 mM at indicated time points. To induce DNA damage, cells were treated with etoposide (MP Biomedicals) at a concentration of 10 μM for 24 hours.

Generation of *in vitro* neurons First, isogenic Rett Syndrome and wild type human induced pluripotent stem cells (hiPSCs) were derived from Rett patient fibroblasts as described previously (Ohashi *et al.*, 2018). The CDKL5 lines were a generous gift from Allyson Muotri and are described elsewhere [29]. hiPSCs were maintained on plates coated with matrigel (Corning) in mTeSR1 (StemCell Technologies) until 80% confluency. Neural progenitor cell (NPC) fate was induced using StemDiff manufacturer's protocol. In our hands, this approach generates a uniform population of immature cortical neurons, as described previously (Patterson et al., 2012). EGF and FGF were removed from the media, and the cells were cultured in DMEMF12 supplemented with N2 and B27 (Thermo Fisher).
