# Supplementary material for: Probing DNA damage in Rett syndrome neurons uncovers a role for MECP2 regulation of PARP1
Source: Stem Cell Reports. 2025 Sep 25;20(10):102645. doi: 10.1016/j.stemcr.2025.102645 (PMC12790717; doi:10.1016/j.stemcr.2025.102645)
Supplement: Document S2. Article plus supplemental information [file mmc4.pdf]

Stem Cell Reports
Article
OPEN ACCESS

Probing DNA damage in Rett syndrome neurons uncovers a role for MECP2 regulation of PARP1

A. Morales,[1,2] E. Korsakova,[1,2,4,5] N. Mansooralavi,[1,4] A. Ravikumar,[2] G. Rivas,[2] P. Soliman,[2,4,5] L. Rodriguez,[2,4,5] T. McDaniel,[2] A. Lund,[2] B. Cooper,[2] A. Bhaduri,[4,5,6] and W.E. Lowry[1,2,3,4,5,7,*]
[1]Molecular Biology Institute, UCLA, Los Angeles, CA 90095, USA
[2]Department of Molecular Cell and Developmental Biology, UCLA, Los Angeles, CA 90095, USA
[3]Division of Dermatology, DGSOM, UCLA, Los Angeles, CA 90095, USA
[4]Broad Center for Regenerative Medicine, UCLA, Los Angeles, CA 90095, USA
[5]Jonsson Comprehensive Cancer Center, UCLA, Los Angeles, CA 90095, USA
[6]Department of Biological Chemistry, DGSOM, UCLA, Los Angeles, CA 90095, USA
[7]Lead contact
*Correspondence: blowry@ucla.edu
https://doi.org/10.1016/j.stemcr.2025.102645

SUMMARY

Methyl-CpG-binding protein 2 (MECP2)/Rett syndrome is characterized by a postnatal loss of neurophysiological function and regression of childhood development. While Rett neurons have been described as showing elevated senescence and P53 activity, here we show that molecular and physiological dysfunction in neurons lacking MECP2 is triggered by elevated DNA damage. Using human induced pluripotent stem cell (hiPSC)-derived isogenic lines, we find that MECP2 directly interacts with members of the DNA repair machinery, including PARP1. Here, we present evidence that MECP2 also regulates PARP1 activity, and restoration of PARP1 activity in MECP2-null neurons can reverse DNA damage, senescence, dendritic branching defects, and metabolic dysfunction. These data from a human disease-in-a-dish model system support the notion that dysfunction in Rett syndrome neurons could be caused by changes in PARP activity.

INTRODUCTION

The disruption of the methyl-CpG-binding protein 2 (MECP2), encoded on the X chromosome, is known to cause a severe neurodevelopmental disease called Rett syndrome (Lewis et al., 1992; Quaderi et al., 1994). MECP2 has been described as both a transcriptional stimulator and inhibitor, a regulator of RNA splicing, and a regulator of DNA methylation or reader of methylation (Fuks et al., 2003; Nan et al., 1998; Tanaka et al., 2014). Moreover, loss of MECP2 has been shown to lead to a variety of cell physiological defects such as mitochondrial permeability, diminished dendritic branching, altered electrophysiological activity, and changes in nuclear and nucleolar size, etc. (Dani et al., 2005; Dastidar et al., 2012; Degano et al., 2014; Tropea et al., 2009; Zhou et al., 2006). Despite all these interesting observations, it is still not clear which of these are relevant for human Rett syndrome patients, which are direct consequences of loss of MECP2 function, nor which should serve as proxies for experimentally targeting and developing therapeutic strategies.

A large number of studies have attempted to find gene expression changes associated with loss of MECP2 in neurons both *in vivo* and *in vitro* (Johnson et al., 2017; Liu et al., 2024; Maxwell et al., 2013; Pecorelli et al., 2013; Tanaka et al., 2014; Vacca et al., 2016). These studies have implicated changes in expression of synaptic genes, metabolic genes, long genes, and specific targets such as brain-derived neurotrophic factor (BDNF) and insulin-like growth factor (IGF) (Chang et al., 2006; Chen et al., 2003, 2015; Klose et al., 2005; Zhou et al., 2006). Besides BDNF and IGF, very few of the transcriptional changes identified in these studies are ubiquitously identified as differentially expressed genes. Instead, one typically finds similar changes in ontological categories of genes to be consistently changed, such as synaptic or metabolic genes (Larimore et al., 2013; Zhou et al., 2006). This observation is interesting in light of previous claims that MECP2 acts as a transcription factor regulating particular genes. Instead, others have argued that perhaps elevated cell stress due to loss of MECP2 leads to physiological changes in neurons driving the etiology of dysfunction in Rett syndrome (Alessio et al., 2018; Ohashi et al., 2018; Squillaro et al., 2010).

Studies with our *in vitro* model of Rett syndrome indicated that specifically postmitotic neurons lacking MECP2 show defects in dendritic branching coincident with induction of p53 and cellular senescence (Ohashi et al., 2018). Importantly, blocking senescence by P53 inhibition restored dendritic branching in Rett-syndrome-patient-derived neurons (Ohashi et al., 2018; Samarasinghe et al., 2021). These results were consistent with those of the Galderisi group who also showed senescence phenotypes in various loss-of-function MECP2 models in human and murine cells (Alessio et al., 2018; Squillaro et al., 2008, 2010, 2019). Furthermore, a more recent study demonstrated that overexpression of MECP2 rescues brain function and abrogated senescence in a mouse model of

Published by Elsevier Inc. on behalf of International Society for Stem Cell Research.
This is an open access article under the CC BY license (http://creativecommons.org/licenses/by/4.0/).

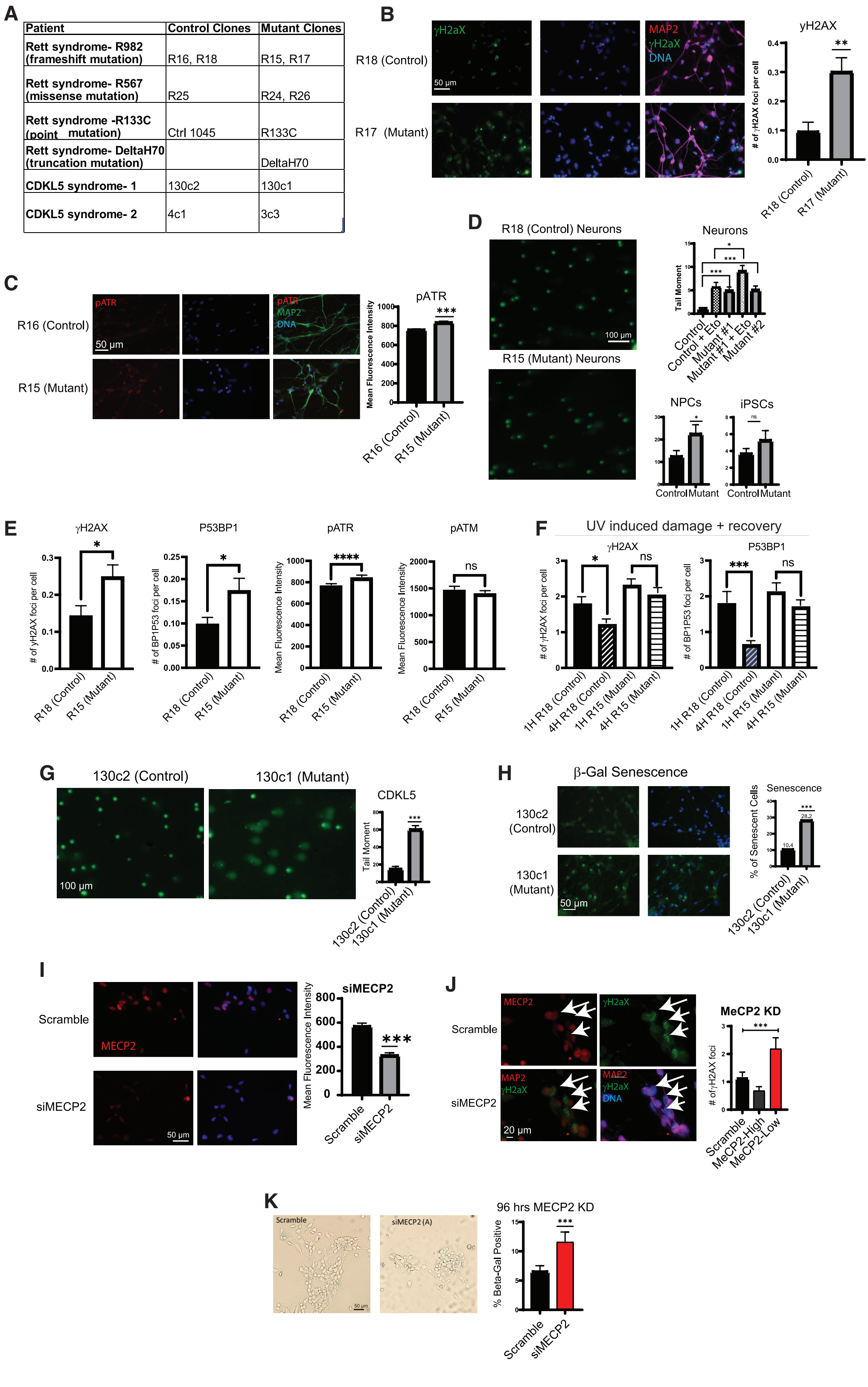

(legend on next page)

age-related cognitive decline (Huang et al., 2021). Thus, while several groups have connected loss of MECP2 function with neuronal senescence, the primary trigger of the stress that leads to senescence has not been established.

Several studies have shown that neurons lacking MECP2 show elevated γH2AX foci, a sign of DNA damage, and have suggested various causes of this phenotype. One more recent study pointed to the possibility that the increased γH2AX is due not necessarily because of increased DNA damage but instead due to larger γH2AX foci as a result of loss of chromatin compaction when MECP2 function is suppressed (Okumura et al., 2025). Here, we present evidence for DNA damage as a trigger of senescence and uncover a role for a PARP1/MECP2 interaction in dysfunction in Rett syndrome.

RESULTS

To discover the trigger for dysfunction in MECP2-null neurons, we created an isogenic *in vitro* system to model how the loss of MECP2 impacts development of human neural cell types by exploiting reprogramming of patient fibroblasts to a pluripotent state to create human induced pluripotent stem cells (hiPSCs) and differentiation toward particular neural lineages (neural progenitor cells [NPCs] and interneurons) (Klose et al., 2005). A summary of patient-derived lines and nomenclature is provided in Figure 1A. Isogenic comparisons of neurons with and without MECP2 showed that loss of MECP2 is correlated with increased DNA damage, senescence, and elevated P53 activity. In addition, the inhibition of P53 pathways reversed most of the Rett neuron phenotypes, indicating the importance of P53 in dysfunction in Rett syndrome (Ohashi et al., 2018). Here, we sought to define the primary trigger of induction of P53 activity that appears to cause downstream neuronal dysfunction in Rett neurons. Known P53 activators include DNA damage, dysfunction of mitochondria or ribosomes, ROS induction, telomere shortening, etc. We were unable to find consistent differences in ROS levels or telomere length between neurons with and without MECP2 (Ohashi et al., 2018), so we asked whether induction of DNA damage or mitochondrial dysfunction in human neurons can drive phenotypes akin to those found after deletion of MECP2.

We first assessed DNA damage in hiPSC-derived neurons via staining for activated ATR, along with γ-H2AX in neurons lacking MECP2. Both of these markers were higher in the absence of MECP2, indicative of elevated DNA damage (Figures 1B and 1C). To determine the extent of physical damage to the genome, we performed an alkaline COMET assay in neurons, which is known to reliably detect single-strand DNA breaks. In fact, neurons, NPCs, and hiPSCs from the isogenic Rett lines described in Figures 1A and 1D showed that loss of MECP2 correlates with elevated DNA damage in both neurons and neural progenitor cells. To distinguish between single- vs. double-strand breaks (SSBs vs. DSBs), we stained neurons for markers of both types of damage on the same cultured neurons. Quantification showed that both γ-H2AX and P53BP1 were elevated in the absence of MECP2 (Figure 1E). In addition, activated ATR was elevated in

Figure 1. DNA damage is strongly associated with loss of MECP2 in isogenic neurons *in vitro*
(A) Table summarizing Rett and CDKL5 syndrome human cell lines derived from patient fibroblasts.
(B) Immunostaining neurons for γ-H2AX or (C) phospho-ATR (pATR), DNA damage response proteins, and neuronal marker MAP2, images taken at 40X. Quantification of mean fluorescence intensity for pATR and puncta per cell for γ-H2AX is shown in the right (n = 100 cells per condition).
(D) Comet assay was used to measure the extent of DNA damage in *in vitro* neurons with or without MECP2 mutant allele. Etoposide, an inhibitor of topoisomerase II, was used as a positive control to induce DNA damage. Images taken at 10X with quantification of tail moment of neurons, NPCs, and human induced pluripotent stem cells (hiPSCs) are shown in the right (n = 100 cells per condition).
(E) Immunostaining for markers of single- and double-strand DNA damage in neuronal cultures with and without MECP2.
(F) DNA repair assay using UV irradiation to induce DNA damage. Cells were irradiated and then fixed after 1 or 4 h. At 1 h, there is extensive damage in both wild-type (WT) and mutant neurons. After 4 h, WT neurons show diminished damage consistent with effective DNA repair.
(G) Comet assay was conducted in *in vitro* neurons with and without CDKL5 allele, and images taken at 10X with quantification of tail moment are shown in the right. (n = 100 cells per condition).
(H) Senescence activity assay on CDKL5 mutant neurons. Data shown are representative of two independent experiments.
(I) WT neurons were treated with siRNA targeting MECP2 for 72 h. Immunostaining with MECP2 shows extent of protein knockdown (n = 100 per condition).
(J) Immunostaining of WT neurons after treatment with small interfering RNA (siRNA) targeting MECP2 for MECP2 and γ-H2AX puncta per cell is on the right. (n = 90 cells per condition).
(K) Senescence activity assay on WT neurons after treatment with siRNA targeting MECP2. (n = 150 cells per condition). Statistical significance for immunostaining experiments was calculated using Student's t test. Statistical significance for senescence experiments was calculated using chi-squared test (*p < 0.05, **p < 0.01, ***p < 0.001). Data shown for each panel are representative of at least two independent experiments.

A

Pathway Analysis of In vitro 13C-Glucose Tracing Metabolomics Experiment

Metabolic Pathway	Total	Expected	Hits	Raw p	-log (p)	Holm adjust	FDR	Impact
Citrate cycle (TCA cycle)	20	0.11613	5	2.55E-08	7.593	2.1444E-06	2.14E-06	0.21156
Alanine, aspartate and glutamate metabolism	28	0.16258	5	1.59E-07	6.7985	0.000013199	6.68E-06	0.2476
Pyruvate metabolism	22	0.12774	4	3.67E-06	5.4352	0.00030105	0.000103	0.23794
Arginine biosynthesis	14	0.08129	3	4.78E-05	4.3206	0.0038719	0.001001	0.11675
Butanoate metabolism	15	0.087097	3	5.96E-05	4.2249	0.0047662	0.001001	0
D-Glutamine and D-glutamate metabolism	6	0.034839	2	0.000444	3.3522	0.035109	0.006222	0.5
Glyoxylate and dicarboxylate metabolism	32	0.18581	3	0.000618	3.2091	0.048195	0.007415	0

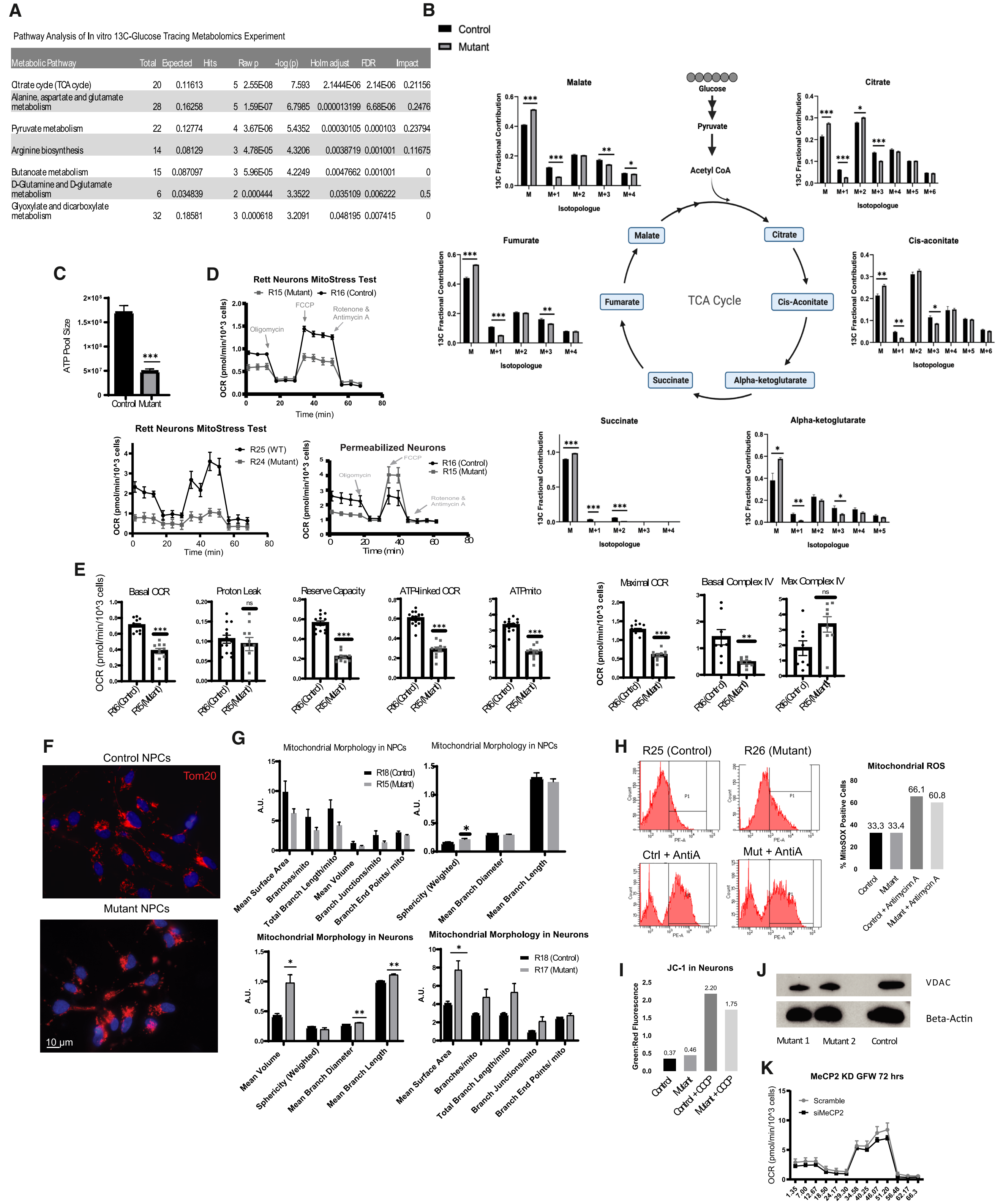

(legend on next page)

mutant neurons but activated ATM was not (Figure 1E). Together, these data suggest that both single- and double-strand DNA damage are elevated in mutant neurons, while only the single-strand repair pathway is activated.

To determine if loss of MECP2 leads to diminished DNA repair activity, we treated WT and MUT neurons with 45 ms of UV irradiation and then fixed them 1 or 4 h later. Staining for markers of both SSBs and DSBs highlighted SSBs as elevated in the absence of MECP2 (Figure 1F). While both wild-type and mutant neurons showed a strong increase in DNA damage in response to UV, neurons lacking MECP2 showed elevated γ-H2AX and P53BP1 foci 4 h after treatment, while wild-type neurons showed evidence of repair, namely reduced γ-H2AX and P53BP1 foci (Figure 1F). We also stained for pATR and pATM in these experiments to determine whether SSB or DSB repair pathways are affected by loss of MECP2. Together with the result of the Alkaline COMET assay, these data are consistent with the notion that loss of MECP2 leaves neurons with increased DNA single- and double-strand DNA breaks potentially due to diminished DNA repair activity.

To determine if elevated DNA damage is potentially a trigger of Rett syndrome phenotypes irrespective of the nature of the mutation, we also assessed isogenic neurons from hiPSCs derived from patients with Rett syndrome caused by mutations in CDKL5 (Fuchs et al., 2014; Jagtap et al., 2019; Loi et al., 2020; Negraes et al., 2021). CDKL5 has previously been implicated in DNA damage and repair, as well as mitochondrial dysfunction (Loi et al., 2020). Here, we found that neurons lacking CDKL5 expression displayed elevated increased tail length with the COMET assay (Figure 1G, left). In addition, as described extensively, the CDKL5 neurons also showed elevated neuronal senescence (Figure 1H). Therefore, it is possible that the phenotypes observed in Rett caused by mutations in either MECP2 or CDKL5 could be triggered by elevated DNA damage.

The fact that elevated DNA damage correlated with loss of MECP2 does not by itself prove that loss of MECP2 leads directly to increased DNA damage nor does it discern whether loss of MECP2 function directly increases the number of breaks or inhibits DNA repair. In addition, this phenotype could be an indirect consequence of loss of MECP2 downstream of other defects. To determine whether DNA damage is a primary consequence of loss of MECP2 in human neurons, we employed RNA interference (Figure 1I). After suppressing MECP2 expression for 72 h by RNA interference (RNAi), we were able to observe an elevation of γ-H2AX foci (Figure 1J) and senescence (Figure 1K). These experiments identified DNA damage as an early consequence of loss of MECP2 function.

Senescence and P53 activation can also be due to mitochondrial dysfunction (Lapasset et al., 2011; Prigione et al., 2010; Rohani et al., 2014; Squillaro et al., 2019). We began by measuring metabolism in neurons lacking MECP2 by metabolomics, glucose tracing, and Seahorse analysis. Metabolic tracing showed that C-13-Glucose produced TCA metabolites at a much lower level in MECP2-null neurons (Figure 2A). Plotting the fractional contribution of glucose carbons into TCA metabolites shows diminished TCA cycle activity (Figure 2B). Metabolomics for total ATP levels also showed evidence of decreased TCA cycle output in MECP2 null neurons (Figure 2C). Measuring oxygen consumption with a Seahorse assay showed that MECP2 mutant neurons from two distinct Rett syndrome patients are defective at oxygen consumption, a readout of TCA cycle activity (Figure 2D). Further analysis of Seahorse data from these experiments

Figure 2. Rett neurons exhibit metabolic dysfunction *in vitro*

(A) Pathway enrichment analysis of UC13-labeled glucose metabolomics with *in vitro* experiments.

(B) Lack of MECP2 in patient-derived neurons leads to a decreased incorporation of UC13-labeled glucose into the citric acid cycle (n = 3 wells per condition).

(C) Decreased ATP metabolite pool size in Rett neurons from metabolomics experiment (n = 3 wells per condition).

(D) Oxygen consumption rate (OCR) and complex-IV-specific activity (bottom) are decreased in Rett neurons as detected by the Seahorse assay (n = 10 wells per condition).

(E) Quantification of Seahorse experiment parameters in Rett neurons (n = 10 cells per condition).

(F) Immunostaining Rett NPCs for mitochondrial marker Tom20.

(G) Quantification of mitochondrial morphology parameters of Rett NPCs and neurons (n = 50 cells per condition).

(H) Quantification of mitochondrial reactive oxygen species in Rett neurons (n = 500 events per condition).

(I) Analysis of mitochondrial permeability using JC-1 dye where an increase in green to red ratio indicates increased membrane permeability (n = 500 events per condition).

(J) Rett neurons were assayed by western blot to assess changes in mitochondrial mass as indicated by mitochondrial protein VDAC.

(K) OCR measured for neurons transfected with siMECP2 for 72 h (n = 5 wells per condition) Data shown are representative of three independent experiments. Statistical significance was calculated using Student's t test (*$p < 0.05$, **$p < 0.01$, ***$p < 0.001$). Metabolomics (Control = R16, Mutant = R17), Seahorse (Control = R16, Mutant = R15), Tom20 NPCs (Control = R18, Mutant = R15), Tom20 Neurons, MitoSOX, and JC-1 (Control = R18, Mutant = R17), VDAC (Control = R18).

showed that these same MECP2 neurons from two patients had a reduced ATP consumption rate relative to isogenic neurons expressing MECP2 (Figure 2E).

In some pathologies, mitochondrial dysfunction can be ascribed to changes in mitochondrial mass, complexity, or morphology (Prigione et al., 2010). We measured the volume and morphology of mitochondria by high-resolution microscopy (Figures 2F and 2G) and found that MECP2-null neurons showed increased volume and surface area of mitochondria, a phenotype previously associated with senescent cells (Figure S1A). On the other hand, we measured mitochondrial reactive oxygen species (Figure 2H), permeability (Figures 2I and S1B), and expression of key proteins (Figure 2J) but failed to find differences between neurons with or without MECP2 expression. Finally, we investigated whether metabolic dysfunction is also a primary response to loss of MECP2. To do this, we employed RNAi approach to acutely suppress MeCP2 expression (Figure 1H) and found that mitochondrial activity was not significantly changed after knockdown (Figure 2K). Together, these data lead to important questions about how mitochondrial function can be influenced by the loss of a nuclear-localized protein like MECP2.

Because previous experiments identified DNA damage as a first consequence of loss of MECP2 function, we sought to assess whether directly targeting DNA damage in wild-type human neurons could phenocopy the loss of MECP2. Inducing DNA damage by etoposide treatment diminished both the oxygen consumption rate (OCR) and TCA metabolism (Figures 3A and 3B). Conversely, deliberately inducing metabolic dysfunction in wild-type neurons did not increase DNA damage (Figure 3C), consistent with the notion that metabolic defects are a result of DNA damage and not a driver of Rett-syndrome-like phenotypes. Therefore, we probed for a role for MECP2 in DNA damage and/or repair.

To identify potential interactions between MECP2 and the DNA repair machinery, we performed immunoprecipitation coupled with mass spectrometry. We carried out IP with an MECP2 antibody with nuclei from two wild-type neuronal lines (R16/R18) and used mass spectrometry to characterize interacting proteins (Figures 4A and 4B). Detection showed 250–350 potential interactors with MECP2 antibody versus samples performed without antibody (Table S1). Between neurons from both cell lines, 130 proteins were shared (Figure 4C), a highly significant overlap. We also carried out the same procedure with nuclei from neurons lacking MEPC2 to eliminate non-specific interactions (Figure S2A). An ontological analysis of authentic interactors highlighted categories of genes such as gene expression, nucleosome organization, and transcription, mostly due to a large number of members of the BAG/BAF complex including at least one member from each of the SMARCA/B/C/D/E families (Figure 4D) (Harikrishnan et al., 2005; Hu et al., 2006). In addition, categories of genes such as nucleotide excision repair and DSB repair also came out of this analysis due to the combination of BAF complex proteins with PARP1. The BAF complex is known to open chromatin to facilitate DNA repair (Harikrishnan et al., 2005; Singhal et al., 2010), and PARP1 is a well-established mediator of DNA repair through its ability to PARylate proteins required for DNA repair (Singhal et al., 2010).

We also mined recent data from the Jaenisch group that performed mass spectrometry on affinity-purified MECP2 interactors from wild-type neurons that found roughly 1,000 proteins (Figure 4E) (Liu et al., 2024). Overlapping these lists of interactors identified in our experiments yielded 76 proteins as interactors of MECP2 across the two studies with distinct methodology. Included in this list are members of the BAF complex, ribosomal proteins, and several DNA repair pathway proteins such as XRCC5, DDB1, TOP1, and PARP1 (Figure 4F). The Jaenisch study also used several tagged MECP2 mutants as a bait, and surprisingly only a few direct interactions appeared to be affected (Liu et al., 2024). For those described here, such as TOP1, XRCC5, and PARP1, the mutations did not appear to prevent binding to MECP2 (Figure S2C). This raises questions about the nature of the interaction between these proteins and MECP2 and why mutations in MECP2 lead to phenotypes in neurons but do not prevent protein-protein binding.

Finally, we mined data in OpenCell (Figure S4B) (Kobayashi et al., 2022), a database summarizing results from a study that used Crispr/Cas9 to introduce peptide tags into individual genes of interest to facilitate both subcellular localization and physical interactions through tandem mass spectrometry pull-down (Figure S4B). MECP2 was one of 1,306 tagged genes in the database. While this study was carried out in HeLa cells, several of the proteins we found to interact with MECP2 were related to DNA repair (Figure S4B), such as PARP1 and NUCKS1(Maranon et al., 2020; Parplys et al., 2015; Sanchez et al., 2022).

From our pull-down, the Jaenisch pull-down, and the OpenCell pull-down (Kobayashi et al., 2022), a particularly notable interaction for MECP2 was with PARP1, an enzyme that adds a poly-ADP-ribose moiety to proteins and is known to have a key role in both DNA repair and transcription. PARP1 has been shown to regulate MECP2 through PARylation to regulate genome structure (Becker et al., 2016), but PARP1 has not been shown to be regulated by MECP2. We performed a reciprocal experiment to confirm the interaction. We used PARP1 antibody to pull down PARP1 as a bait and perform mass spectrometry to identify interacting proteins. We were able to identify 360 proteins compared to minus antibody condition, and MECP2 was

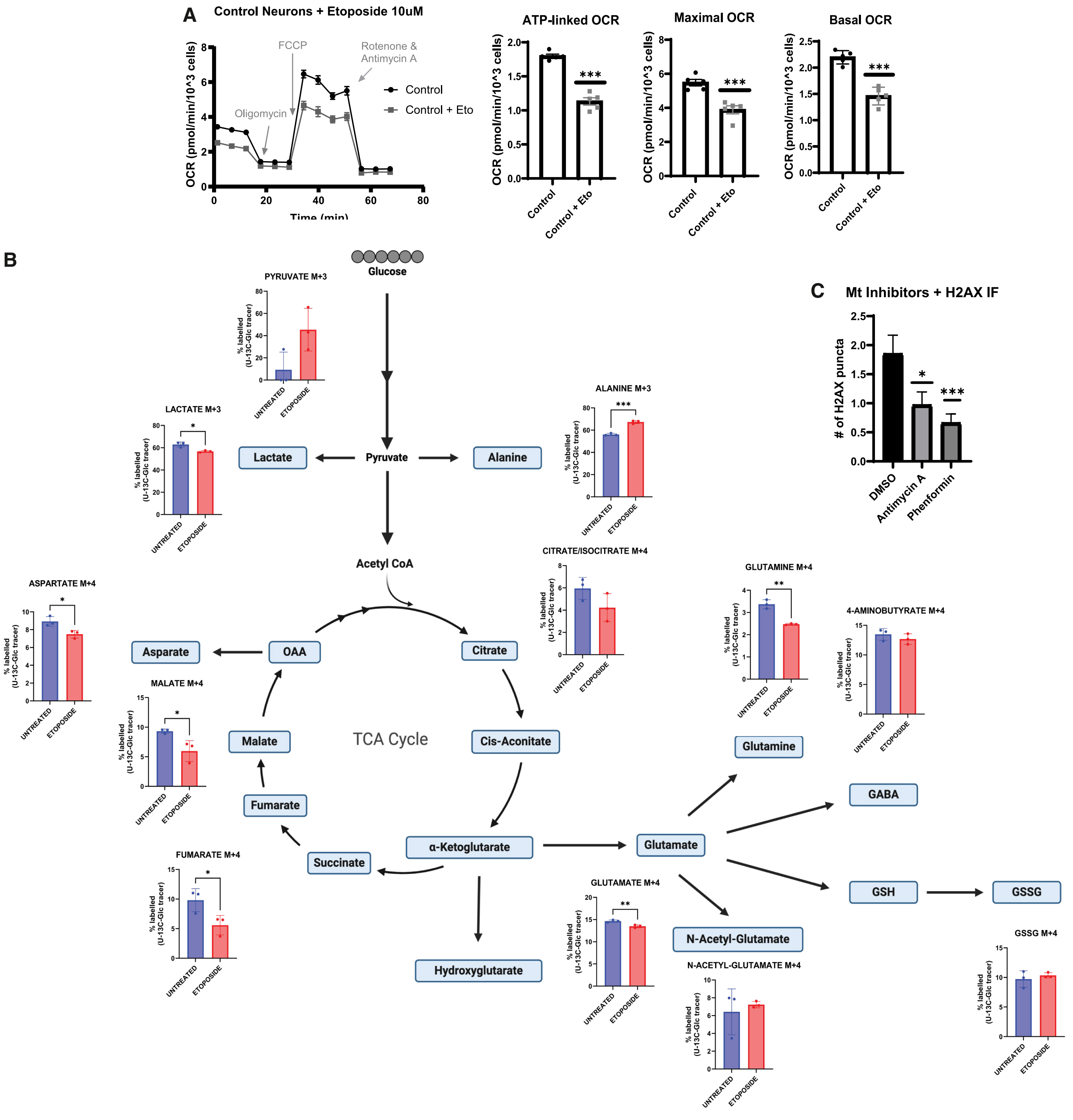

Figure 3. DNA damage is a primary response to loss of MeCP2

(A) Analysis of OCR by Seahorse assay on WT neurons treated with etoposide (*n* = 5 wells per condition).
(B) UC13-glucose tracing metabolomics experiment of WT neurons treated with etoposide (10 μM) for 24 h (*n* = 3 wells per condition).
(C) Quantification of immunostaining experiment of WT neurons treated with antimycin A (1 μM) or phenformin (20 μM) for 24 h for γ-H2AX puncta per cell. (*n* = 150 cells per condition).
Statistical significance for Seahorse and immunostaining experiments was calculated using Student's t test (**p* < 0.05, ***p* < 0.01, ****p* < 0.001). Data shown for each panel are representative of at least two independent experiments.

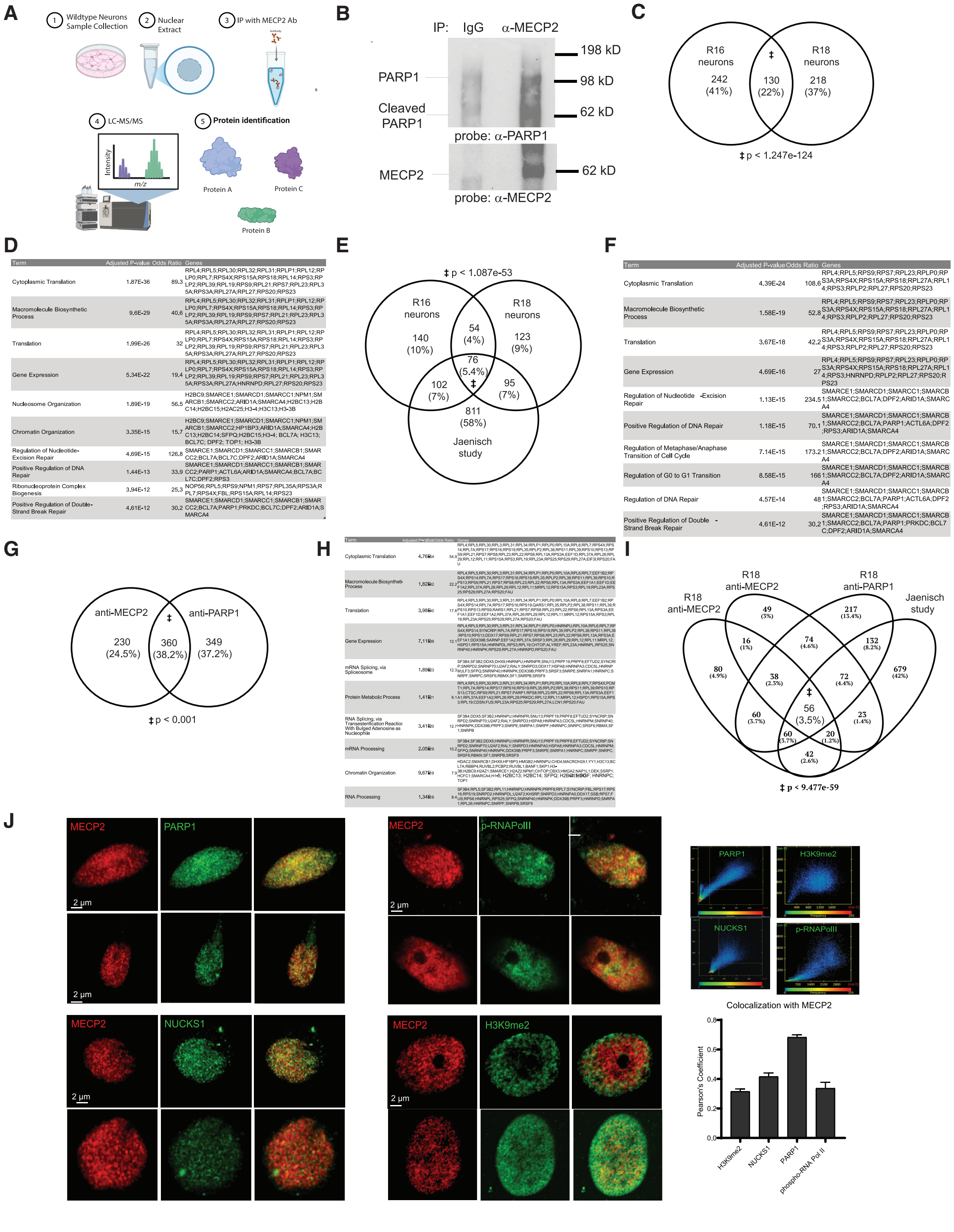

Figure 4. MECP2 interacts with PARP1 in human neurons

(A) Diagram outlining the co-immunoprecipitation (co-IP) mass spectrometry (MS) experiments to identify protein-protein interactions of MECP2 and PARP1 using nuclear lysate from human neurons.

(B) MECP2 was immunoprecipitated and then western blot was performed with antibody against PARP1. As a control, pull-down with IgG did not precipitate MECP2 or PARP1.

(C) Venn diagram showing shared protein interactors of MECP2 in both R16 and R18 WT neurons and calculated *p* value of the overlap.

(D) Gene ontology (GO) analysis of shared protein interactors from (C).

(legend continued on next page)

among the top (Figure 4G; Table S2). Gene ontological analysis of the proteins identified shared some similarity with that of MECP2 interactors including categories related to transcription, chromatin regulation, and DNA repair (Figure 4H). Finally, we overlapped all the interactors across all described data and still identified 56 shared interacting proteins, suggesting that MECP2 and PARP1 potentially form a highly enriched complex in human neurons (Figure 4I).

We then used confocal microscopy to determine whether MECP2 co-localizes in human neurons with PARP1, and other proteins previously proposed to interact with MECP2 (Figure 4J). This approach showed that MECP2 also co-localized with the DNA repair proteins PARP1 and NUCKS1 to a very high degree, with over 0.5–0.6 Pearson's correlation coefficient across multiple wild-type neurons (Figure 4J, right). As a measure of the specificity of these interactions, we also attempted to co-localize MECP2 with several other nuclear proteins related to heterochromatin or subnuclear domains (Figure 4I), none of which overlapped as significantly as PARP1. Together, these three lines of evidence indicate a direct interaction between PARP1 and MECP2, along with other proteins related to DNA repair in the nucleus.

We next sought to determine whether MECP2 and PARP1 functionally interact. PARP1 activity can be measured via ELISA assay to detect PARylation of a protein substrate. We assessed PARP activity in neurons with and without MECP2 and consistently found lower levels of activity in the absence of MECP2 across multiple genetic backgrounds representing distinct MECP2 mutations and in MECP2-null rat brain (Figure 5A), suggesting a functional connection between the two proteins in various contexts associated with Rett-like phenotypes. It is worth noting that MECP2 has been shown previously to be PARylated by PARP1 (Becker et al., 2016; Loi et al., 2020).

PARP1 inhibitors are used to block DNA repair leading to catastrophic DNA damage in cancer cells and have become an effective treatment adjuvant (Penning, 2010). In addition, it is known that PARP1 inhibition with olaparib can induce cellular senescence in various cell types as measured by β-galactosidase assay (Penning, 2010). Therefore, we sought to determine whether PARP inhibition in normal neurons can phenocopy the outcome of loss of MECP2. First, we titrated to find an effective dose of olaparib to decrease PARP1 activity in human neurons (Figure 5B). We then measured DNA damage and senescence in wild-type neurons treated with olaparib and found that PARP inhibition can indeed drive DNA damage and senescence (Figures 5C and 5D), in a similar fashion to loss of MECP2 by mutation or siRNA. In addition, PARP1 inhibition by olaparib diminished oxygen consumption and ATP production as measured by Seahorse (Figure 5E), and metabolomics showed a similar phenotype on TCA cycle activity as shown for loss of MECP2 (Figure 5F).

To further probe for a functional interaction between PARP1 and MECP2, we sought to test whether stimulation of PARP1 activity in neurons lacking MECP2 can reverse the phenotypes caused by loss of MECP2. PARP1 is known to be stimulated *in vivo* and *in vitro* by elevated levels of NAD, a key co-factor for its enzymatic activity [49]. We first assayed for an effective concentration for PARP1 stimulation by NAD and found that addition of 10 mM NAD can dramatically stimulate PARP1 activity in MECP2 mutant neurons but did not alter levels of PARP1 activity in wild-type neurons (Figure 6A). We then assessed the effect of 10 mM NAD on MECP2-null neurons to determine whether PARP1 stimulation could diminish Rett-syndrome-relevant defects. NAD treatment significantly decreased DNA damage as measured by both γ-H2AX staining (Figure 6B) and COMET assay (Figure 6C). Because MECP2-null neurons treated with NAD showed less DNA damage, we assessed senescence in these cells under PARP1 stimulation. Indeed, by fluorescent β-gal activity assay, we found that NAD treatment could diminish senescence in MECP2-null neurons (Figure 6D). In addition, these effects of NAD on DNA damage and senescence both were blocked by the addition of Olaparib, demonstrating that the effect of NAD was indeed due to upregulation of PARP1 (Figures 6B–6D).

Using RNA sequencing (RNA-seq) from MECP2 wild-type vs. mutant neurons, we identified many genes as up- or downregulated in the mutant (Figure 6E). Ontological analysis of the DEGs showed that those that were upregulated

(E) Venn diagram comparing multiple proteomic datasets consisting of two control cell lines with MECP2 pull-down and an independent MECP2 proteomic experiment from the Jaenisch group, along with a calculated *p* value of the overlap between the three datasets.
(F) Gene ontology (GO) analysis of shared protein interactors from (E).
(G) Venn diagram of interactors from both MECP2 and PARP1 IP-mass spectrometry and calculated *p* value of the overlap.
(H) Summary of gene ontology of the overlapping interactors from both MECP2 and PARP1 experiments.
(I) Venn diagram of interactors of MECP2 in two WT lines, the Jaenisch equivalent experiment, and with PARP1 IP-mass spec.
(J) Confocal microscopy of WT neurons to observe degree of colocalization between MeCP2 and PARP1, NUCKS1 phospho-RNA polymerase II, and H3K9me2. Right: scatter plot of pixel intensities across two channels (red: *x* axis and green: *y* axis) for each confocal image. Right: quantification of colocalization between MeCP2 and PARP1, NUCKS1, H3K9me2, and phospho-RNA Pol II based on Pearson's correlation coefficient (PCC) (*n* = 60 images per condition). Venn diagram statistical significance was determined by hypergeometric test.

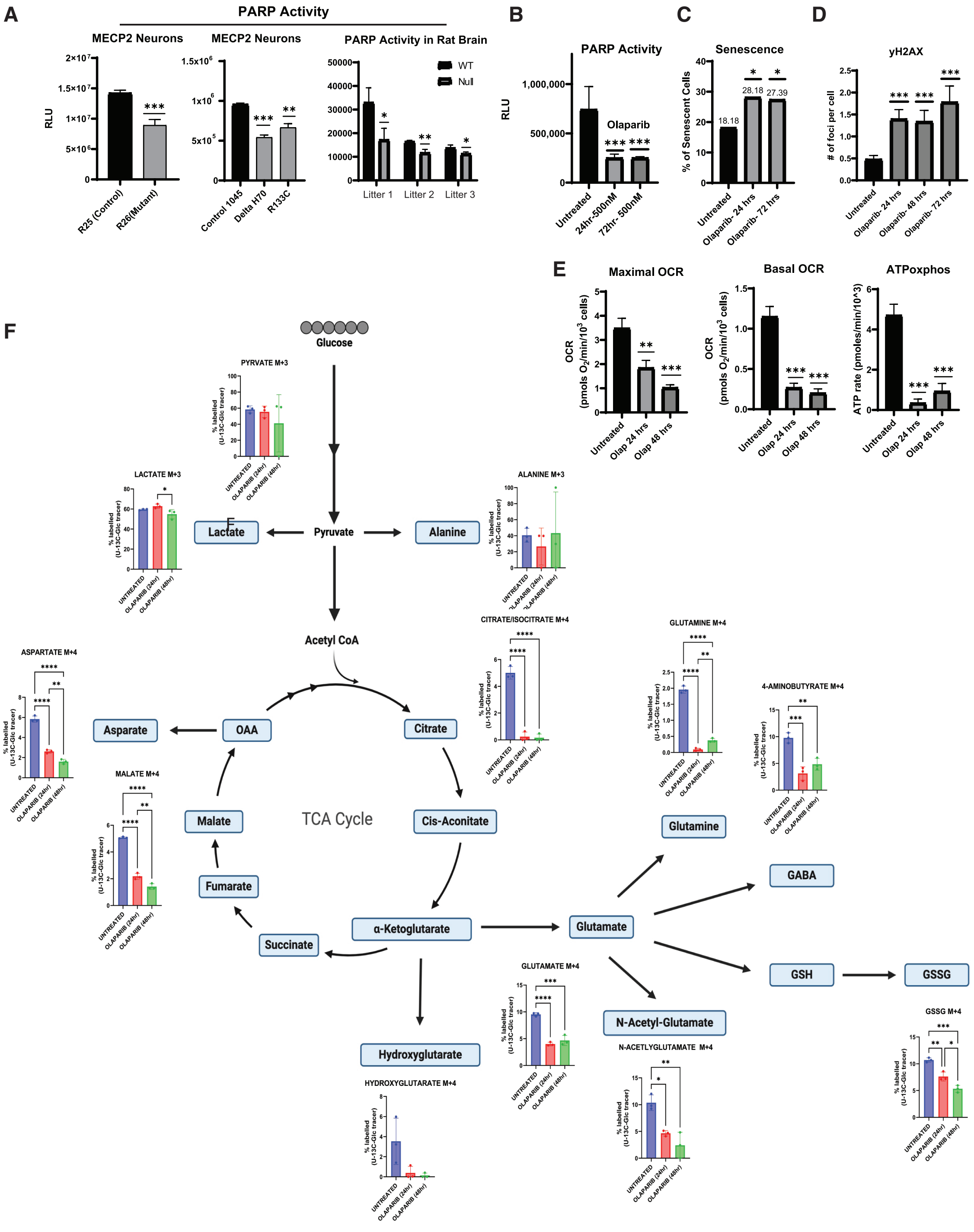

Figure 5. Parp1 activity is depressed in the absence of MECP2 in neurons

(A) Measurement of PARP activity of Rett syndrome neurons and Rett rat model through an ELISA-based plate assay (n = 3 wells per condition).

(B) Measurement of PARP activity of WT neurons after treatment with PARP1 inhibitor olaparib (n = 3 wells per condition).

(C) WT neurons treated with olaparib (500 nM) had increased incidence of senescence as shown in quantification of β-galactosidase staining (n = 150 cells per condition).

(D) Quantification of immunostaining of WT neurons treated with olaparib (500 nM) for γ-H2AX foci (n = 100 cells per condition).

(legend continued on next page)

in the absence of MECP2 were mostly related to DNA damage and repair and that NAD treatment diminished the expression of genes related to DNA damage and repair (Figure 6F). In other words, many of the upregulated genes related to DNA damage and repair such as RAD51, BRCA1, ERCC1, and MRE11 that were induced by loss of MECP2 were suppressed by NAD treatment (Figures 6E and 6F), consistent with the diminished DNA damage shown by γ-H2AX staining and COMET assay.

Perhaps the defining characteristic of Rett syndrome neurons is defects in dendritic branching, which has been described in essentially all models to date both *in vivo* and *in vitro*. Importantly, MECP2 null neurons treated with NAD showed a statistically significant increase in dendritic complexity (Figure 6G). This is a key phenotype thought to mediate many of the electrophysiological manifestations of Rett syndrome, as it is linked to neuronal network activity, and we and others previously defined as a critical feature of the syndrome. Similarly, we showed that mitochondrial function is diminished in the absence of MECP2, but treatment with NAD reversed much of this defect (Figure 6H). Together, the data in Figures 5 and 6 demonstrate that PARP1 activity is potentially important to understand how loss-of-function mutations in MECP2 lead to patient phenotypes and point toward novel interventional approaches.

DISCUSSION

This study sheds new light on the etiology of Rett syndrome by molecular interrogation of individual neurons in isogenic neurons *in vitro*. We find that DNA breaks are a primary trigger for dysfunction in MECP2 neurons and that PARP1 is a key mediator of this effect. While previous efforts have described DNA damage as a phenotype in Rett syndrome models, it was not previously implicated as a driver of phenotypes. The majority of previous studies argue that patient phenotypes occur due to misregulation of gene expression as a consequence of loss of MECP2 function resulting from altered chromatin structure or function or because MECP2 is thought to serve as a transcriptional repressor. Instead, the data presented here point to the potential that patient phenotypes are instead the result of a stress response of neurons to dysfunctional DNA repair, because reversal of the stress response (Ohashi et al., 2018; Samarasinghe et al., 2021) or alleviation of DNA damage by restoration of PARP activity (Figure 6) appears to restore normal function to neurons lacking MECP2.

This finding is potentially relevant to one previous report showing that MECP2 is a PARylation target of PARP1 (Becker et al., 2016). Because MECP2 is an abundant protein in neurons and a target of PARylation, it is tempting to speculate whether other proteins show an increase in PARylation when MECP2 is not present in neurons. While beyond the scope of this study, this hypothesis represents another potential consequence of loss of MECP2. On the other hand, treatment of wild-type neurons with a PARP1 inhibitor (olaparib) generated phenotypes similar to those caused by loss of MECP2, which would suggest that altered PARylation levels, and not altered targeting of PARylation, are responsible for Rett phenotypes in neurons.

It has been argued that senescence may be a cellular adaptation designed to conserve energy required for division and differentiation so the cell can survive when facing stress (Ishikawa, 2006; Sikora, 2013). The Galderisi group has published several studies demonstrating senescence in RTT patient mesenchymal stem cells (MSCs), partially MECP2-silenced human MSCs, MECP2-silenced human neuroblastoma cells, and heterozygous MECP2 mutant mouse mesenchymal stromal cells and neural stem cells (Alessio et al., 2018; Squillaro et al., 2008, 2010, 2019). In these studies, senescence and DNA damage were proposed to be mediated by defects in metabolism that led to elevation of reactive oxygen species, which then triggers DNA damage, leading to elevation of P53 activity and senescence. Here, we show that DNA damage is a primary trigger for defects in MECP2 null neurons and that loss of MECP2 activity appears to affect DNA repair more directly through an interaction with PARP1. In addition, we found that neurons lacking functional CDKL5 have a very similar phenotype as those lacking MECP2. Together, these results suggest that the similarity between Rett (MECP2) and CDKL5 patient symptoms are potentially due to a shared etiology of elevated DNA damage and PARP1 dysfunction. This is interesting in light of data showing that mice lacking functional CDKL5 show increased neuronal senescence and premature aging (Fuchs et al., 2018; Loi et al., 2020), similar to what we have observed in human neurons lacking MECP2.

We previously showed that inhibition of neuronal senescence in MECP2-null neurons is sufficient to restore relatively normal function. However, this approach would not solve the underlying problem causing the senescence,

(E) Seahorse experiment shows decreased mitochondrial OCR of WT neurons after treatment with olaparib (500 nM) (n = 5 wells per condition).
(F) UC13 glucose tracing metabolomics experiment shows decrease in glucose contribution to the TCA cycle and glutaminolysis of WT neurons after treatment with olaparib (500 nM) (n = 3 wells per condition). Statistical significance was calculated using Student's t test and two-way ANOVA for experiments with groups greater than two (*$p < 0.05$, **$p < 0.01$, ***$p < 0.001$). Data shown for each panel are representative of at least two independent experiments.

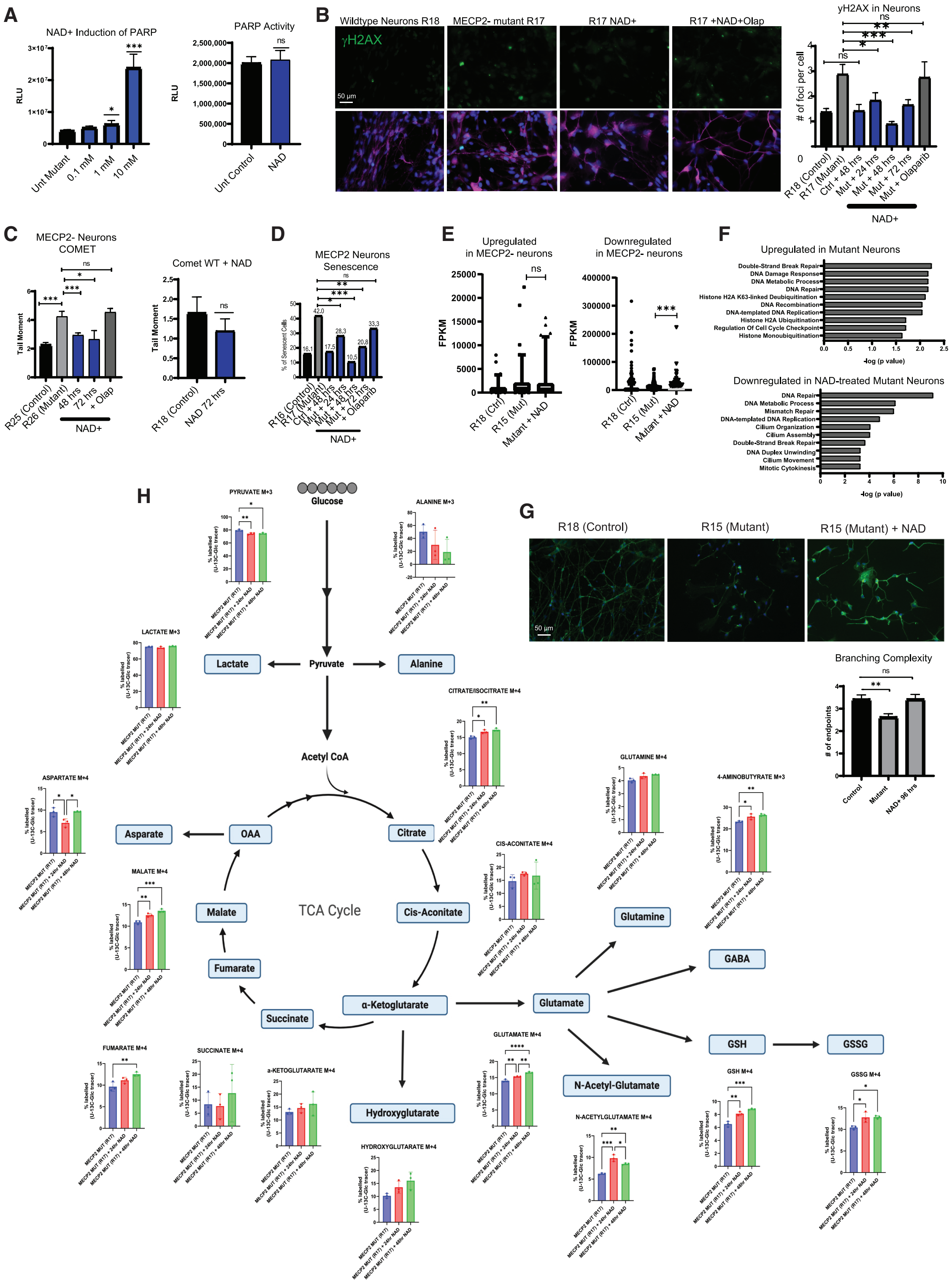

Figure 6. Stimulation of PARP1 activity through NAD supplementation rescues Rett syndrome deficiencies in neurons

(A) Induction of PARP activity, as measured by ELISA-based plate assay, in MECP2 mutant (left) and WT (right) neurons through supplementation of nicotinamide adenine dinucleotide hydrate (NAD) for 24 h (n = 3 wells per condition).

(legend continued on next page)

namely the elevated DNA damage observed in this study. In pursuing a clinical strategy for the treatment of Rett syndrome, one could therefore attempt to promote DNA repair. It is worth noting in our study that supplementing NAD was sufficient to promote PARP1 activity and reverse many of the defects caused by loss of MECP2 function, including DNA damage, senescence, dendritic branching, and metabolism. The activation of P53 pathways in Rett syndrome neurons has parallels in aging, and it is tempting to consider Rett syndrome as a potential early-aging syndrome, as many of the effects of loss of MECP2 overlap with phenotypes that are known to occur in aged neurons such as dendritic defects, metabolic defects, epigenetic instability, lysosomal dysfunction, or senescence. If true, it is interesting that many have proposed NAD supplementation as an anti-aging strategy for chronological aging, which is thought to be associated with NAD deficiency. Perhaps MECP2 syndrome patients could benefit from such supplementation; this will need to be tested in preclinical models such as transgenic Rett/MECP2 rodent models.

This highlights a limitation of the current study. All the data presented here were generated in our *in vitro* stem-cell-derived model. We previously showed that inhibition of senescence with small molecules was sufficient to reverse Rett phenotypes in 2D cultured neurons and then repeated this finding with an organoid model where P53 inhibition restored neuronal network activity. Going forward it will be important to test the same effect described here for PARP stimulation in Rett organoids and transgenic animals lacking MECP2 to further define the clinical relevance for this pathway in the etiology of Rett syndrome.

METHODS

Immunofluorescence and image quantification

Cells grown on coverslips were washed with PBS and fixed with 4% paraformaldehyde (Electron Microscopy Sciences) for 15 min at room temperature. Next, the cells were washed with 0.1% PBST three times and blocked in MAXblock Blocking Medium (Active Motif) for 1 h at room temperature, then incubated overnight at 4°C in the primary antibody. The following primary antibodies were used: rabbit MECP2 (Diagenode C15410052, 1:1,000), mouse phosphor-γH2AX (Millipore 05-636, 1:2,000), rabbit pATR (Abcam ab227851, 1:500), mouse PARP1 (LS Bio LS-C41045, 1:500), mouse NUCKS1 (US Biological 249527, 1:100), mouse H3K9me2 (Abcam ab1220, 1:300), mouse nucleolin (Abcam ab136649, 1:1,000), mouse coilin (Abcam ab11822, 1:2,000), chicken MAP2 (Novus Bio NB300- 213, 1:2,000), mouse pATM (Abcam ab81292, 1:500), mouse SC35 (Abcam ab11826, 1:100), rat phospho-RNA polymerase II (Millipore MABE953, 1:500), and mouse Tom20 (Santa Cruz Biotechnology sc17764, 1:50). Next, the slides were washed three times with 0.1% PBST, and secondary antibody conjugated with Alexa 488, 568, 594, or 647 (1:500, Life Technologies A-21203, A21202, A31571, A-21207) was used, accompanied by DAPI (Invitrogen 1:500). Slides were then washed three times with 0.1% PBST and mounted using Prolong Gold (Invitrogen). Mean fluorescence intensity and/or puncta number per cell were quantified using ImageJ in blind analysis.

β-Galactosidase senescence assay

β-Galactosidase senescence assay was performed using either the Senescence β-Galactosidase Staining Kit from Cell Signaling or CellEvent Senescence Green Detection Kit from Invitrogen following respective manufacturers' protocol. The number of blue/green cells and number of total cells were quantified using the cell counter plugin in ImageJ.

Alkaline comet assay

The alkaline comet assay was performed as previously described (https://doi.org/10.1093/hmg/ddw395), with some minor changes. From a 24-well tissue culture plates (Greiner), cells were then harvested with TrypLE, spun

(B) Left: immunostaining of Rett neurons following treatment with NAD and NAD with olaparib for γ-H2AX. Right: quantification of γ-H2AX foci per cell (n = 100 cells per condition).
(C) Quantification of COMET assay experiment of Rett neurons following stimulation of PARP activity with NAD treatment (n = 100 cells per condition).
(D) Quantification of β-gal galactosidase experiment of Rett neurons following treatment with NAD (n = 150 cells per condition).
(E) DEGs from WT vs. MECP2— neurons bulk RNA-seq, along with treatment with NAD. A statistically significant portion of the genes downregulated by loss of MECP2 were upregulated by NAD treatment.
(F) Ontological analysis of the genes upregulated in MECP2— neurons and those downregulated by NAD.
(G) Measurement of dendritic complexity on Rett neurons following treatment with NAD for 96 h (n = 50 cells per condition).
(H) UC13 glucose tracing metabolomics experiment on mutant neurons shows increased TCA flux after treatment with NAD (n = 3 wells per condition). Statistical significance was calculated using Student's t test two-way ANOVA for experiments with groups greater than two (*$p < 0.05$,**$p < 0.01$, ***$p < 0.001$). Data shown for each panel are representative of at least two independent experiments.

down, and resuspended in 0.5% low-melting-point agarose at 37°C in a 1:10 ratio. Cell suspension was spread onto agarose-coated slides and allowed to polymerize for 20 min in the dark. After agarose solidification, samples were incubated in lysis buffer (10 mM Tris-HCl, pH 10, 2.5 M NaCl, 0.1 M EDTA, 1% Triton X-100) for 2 h. Sorted nuclei from the brain were incubated in modified lysis buffer (1 mM Tris-HCl, pH 10, 2.5 M NaCl, 0.1 M EDTA, 1% Triton X-100) for 1 h. Following removal of lysis buffer, samples were incubated in alkaline running buffer (0.3 M NaOH, 1 mM EDTA) for 30 min and finally electrophoresed at 300 mA for 30 min at 4°C. Cells were stained with Vista Green DNA staining solution (Abcam) for 15 min at room temperature, washed with dH2O, and allowed for agarose to dry overnight. Images were acquired on the Zeiss Axio Imager A2. Images were analyzed using the CometScore 2.0 software.

PARP activity measurements

PARP activity was analyzed from lysed cultured cells using PARP1 Chemiluminescent kit (RnD System) according to manufacturer instructions. Samples were normalized based on protein concentration according to the Pierce BCA Protein Assay (Thermo Fisher). Rat brain samples were provided by Dr. Michelle Olsen, Virginia Tech University. Brains were prepared in cell lysis buffer for Parp1 activity assays.

Co-immunoprecipitation

Nuclear lysate was prepared from human wild-type neurons using the same approach as described above for the brain samples. The lysate was probed with either immunoglobulin G (IgG) or an antibody against MECP2. These were incubated for two hours and then precipitated with protein-A beads. The beads were then run on a western blot and probed with an antibody against PARP1. In another embodiment, the immunoprecipitated was analyzed by mass spectrometry to identify the proteins present.

Human Cell Sourcing

The cell lines used in this study were described in previous publications. They were derived from skin fibroblasts from anonymized human donors, and thus do not constitute human subjects research, and are therefore exempt from IRB oversight.

RESOURCE AVAILABILITY

Lead contact

Requests for further information and resources should be directed to and will be fulfilled by the lead contact, William Lowry (blowry@ucla.edu).

Materials availability

Any materials or cell lines described in this study will be made available upon request.

Data and code availability

RNA-seq data are available at NIH-GEO (GSE301702, GEO Accession viewer). Any other data can be made available upon request.

ACKNOWLEDGMENTS

We would like to acknowledge the various core facilities at UCLA that made this work possible, including Genomics (Department of Pathology) and Flow Cytometry (Jonsson Comprehensive Cancer Center). A.M. was supported by the training program in Neurobehavioral Genetics (NIH). E.K. and N.M. were supported by the BSCRC training program in stem cell biology, sponsored by the Rose Hills Foundation and CIRM. This work was supported by grants from the International Rett Syndrome Foundation (#3904) and the California Institute for Regenerative Medicine (#DISC0014519).

AUTHOR CONTRIBUTIONS

A.M., E.K., N.M., A.R., G.R., P.S., T.M., A.L., and B.C. performed experiments and quantification. A.M. and N.M. assembled the data and generated figures. A.M. and W.E.L. prepared the manuscript.

DECLARATION OF INTERESTS

W.E.L. is a founder and shareholder of Pelage Pharmaceuticals, Sardona Therapeutics, and Cellio Biotechnology. The work presented here was not supported by any of these companies. The data described here were used to generate a filing for patent protection.

SUPPLEMENTAL INFORMATION

Supplemental information can be found online at https://doi.org/10.1016/j.stemcr.2025.102645.

Received: March 4, 2025
Revised: August 21, 2025
Accepted: August 27, 2025
Published: September 25, 2025

REFERENCES

Alessio, N., Riccitiello, F., Squillaro, T., Capasso, S., Del Gaudio, S., Di Bernardo, G., Cipollaro, M., Melone, M.A.B., Peluso, G., and Galderisi, U. (2018). Neural stem cells from a mouse model of Rett syndrome are prone to senescence, show reduced capacity to cope with genotoxic stress, and are impaired in the differentiation process. Exp. Mol. Med. *50*, 1. https://doi.org/10.1038/s12276-017-0005-x.

Becker, A., Zhang, P., Allmann, L., Meilinger, D., Bertulat, B., Eck, D., Hofstaetter, M., Bartolomei, G., Hottiger, M.O., Schreiber, V., et al. (2016). Poly(ADP-ribosyl)ation of Methyl CpG Binding Domain Protein 2 Regulates Chromatin Structure. J. Biol. Chem. *291*, 4873–4881. https://doi.org/10.1074/jbc.M115.698357.

Chang, Q., Khare, G., Dani, V., Nelson, S., and Jaenisch, R. (2006). The disease progression of Mecp2 mutant mice is affected by the

level of BDNF expression. Neuron *49*, 341–348. https://doi.org/10.1016/j.neuron.2005.12.027.

Chen, L., Chen, K., Lavery, L.A., Baker, S.A., Shaw, C.A., Li, W., and Zoghbi, H.Y. (2015). MeCP2 binds to non-CG methylated DNA as neurons mature, influencing transcription and the timing of onset for Rett syndrome. Proc. Natl. Acad. Sci. USA *112*, 5509–5514. https://doi.org/10.1073/pnas.1505909112.

Chen, W.G., Chang, Q., Lin, Y., Meissner, A., West, A.E., Griffith, E. C., Jaenisch, R., and Greenberg, M.E. (2003). Derepression of BDNF transcription involves calcium-dependent phosphorylation of MeCP2. Science *302*, 885–889. https://doi.org/10.1126/science.1086446.

Dani, V.S., Chang, Q., Maffei, A., Turrigiano, G.G., Jaenisch, R., and Nelson, S.B. (2005). Reduced cortical activity due to a shift in the balance between excitation and inhibition in a mouse model of Rett syndrome. Proc. Natl. Acad. Sci. USA *102*, 12560–12565. https://doi.org/10.1073/pnas.0506071102.

Dastidar, S.G., Bardai, F.H., Ma, C., Price, V., Rawat, V., Verma, P., Narayanan, V., and D'Mello, S.R. (2012). Isoform-specific toxicity of Mecp2 in postmitotic neurons: suppression of neurotoxicity by FoxG1. J. Neurosci. *32*, 2846–2855. https://doi.org/10.1523/JNEUROSCI.5841-11.2012.

Degano, A.L., Park, M.J., Penati, J., Li, Q., and Ronnett, G.V. (2014). MeCP2 is required for activity-dependent refinement of olfactory circuits. Mol. Cell. Neurosci. *59*, 63–75. https://doi.org/10.1016/j.mcn.2014.01.005.

Fuchs, C., Gennaccaro, L., Trazzi, S., Bastianini, S., Bettini, S., Lo Martire, V., Ren, E., Medici, G., Zoccoli, G., Rimondini, R., and Ciani, E. (2018). Heterozygous CDKL5 Knockout Female Mice Are a Valuable Animal Model for CDKL5 Disorder. Neural Plast. *2018*, 9726950. https://doi.org/10.1155/2018/9726950.

Fuchs, C., Trazzi, S., Torricella, R., Viggiano, R., De Franceschi, M., Amendola, E., Gross, C., Calzà, L., Bartesaghi, R., and Ciani, E. (2014). Loss of CDKL5 impairs survival and dendritic growth of newborn neurons by altering AKT/GSK-3beta signaling. Neurobiol. Dis. *70*, 53–68. https://doi.org/10.1016/j.nbd.2014.06.006.

Fuks, F., Hurd, P.J., Wolf, D., Nan, X., Bird, A.P., and Kouzarides, T. (2003). The methyl-CpG-binding protein MeCP2 links DNA methylation to histone methylation. J. Biol. Chem. *278*, 4035–4040. https://doi.org/10.1074/jbc.M210256200.

Harikrishnan, K.N., Chow, M.Z., Baker, E.K., Pal, S., Bassal, S., Brasacchio, D., Wang, L., Craig, J.M., Jones, P.L., Sif, S., and El-Osta, A. (2005). Brahma links the SWI/SNF chromatin-remodeling complex with MeCP2-dependent transcriptional silencing. Nat. Genet. *37*, 254–264. https://doi.org/10.1038/ng1516.

Hu, K., Nan, X., Bird, A., and Wang, W. (2006). Testing for association between MeCP2 and the brahma-associated SWI/SNF chromatin-remodeling complex. Nat. Genet. *38*, 962–967. https://doi.org/10.1038/ng0906-962.

Huang, J.L., Zhang, F., Su, M., Li, J., Yi, W., Hou, L.X., Yang, S.M., Liu, J.Y., Zhang, H.A., Ma, T., and Wu, D.P. (2021). MeCP2 prevents age-associated cognitive decline via restoring synaptic plasticity in a senescence-accelerated mouse model. Aging Cell *20*, e13451. https://doi.org/10.1111/acel.13451.

Ishikawa, F. (2006). Cellular senescence as a stress response. Cornea *25*, S3–S6. https://doi.org/10.1097/01.ico.0000247206.47897.92.

Jagtap, S., Thanos, J.M., Fu, T., Wang, J., Lalonde, J., Dial, T.O., Feiglin, A., Chen, J., Kohane, I., Lee, J.T., et al. (2019). Aberrant mitochondrial function in patient-derived neural cells from CDKL5 deficiency disorder and Rett syndrome. Hum. Mol. Genet. *28*, 3625–3636. https://doi.org/10.1093/hmg/ddz208.

Johnson, B.S., Zhao, Y.T., Fasolino, M., Lamonica, J.M., Kim, Y.J., Georgakilas, G., Wood, K.H., Bu, D., Cui, Y., Goffin, D., et al. (2017). Biotin tagging of MeCP2 in mice reveals contextual insights into the Rett syndrome transcriptome. Nat. Med. *23*, 1203–1214. https://doi.org/10.1038/nm.4406.

Klose, R.J., Sarraf, S.A., Schmiedeberg, L., McDermott, S.M., Stancheva, I., and Bird, A.P. (2005). DNA binding selectivity of MeCP2 due to a requirement for A/T sequences adjacent to methyl-CpG. Mol. Cell *19*, 667–678. https://doi.org/10.1016/j.molcel.2005.07.021.

Kobayashi, H., Cheveralls, K.C., Leonetti, M.D., and Royer, L.A. (2022). Self-supervised deep learning encodes high-resolution features of protein subcellular localization. Nat. Methods *19*, 995–1003. https://doi.org/10.1038/s41592-022-01541-z.

Lapasset, L., Milhavet, O., Prieur, A., Besnard, E., Babled, A., Aït-Hamou, N., Leschik, J., Pellestor, F., Ramirez, J.M., De Vos, J., et al. (2011). Rejuvenating senescent and centenarian human cells by reprogramming through the pluripotent state. Genes Dev. *25*, 2248–2253. https://doi.org/10.1101/gad.173922.111.

Larimore, J., Ryder, P.V., Kim, K.Y., Ambrose, L.A., Chapleau, C., Calfa, G., Gross, C., Bassell, G.J., Pozzo-Miller, L., Smith, Y., et al. (2013). MeCP2 regulates the synaptic expression of a Dysbindin-BLOC-1 network component in mouse brain and human induced pluripotent stem cell-derived neurons. PLoS One *8*, e65069. https://doi.org/10.1371/journal.pone.0065069.

Lewis, J.D., Meehan, R.R., Henzel, W.J., Maurer-Fogy, I., Jeppesen, P., Klein, F., and Bird, A. (1992). Purification, sequence, and cellular localization of a novel chromosomal protein that binds to methylated DNA. Cell *69*, 905–914.

Liu, Y., Flamier, A., Bell, G.W., Diao, A.J., Whitfield, T.W., Wang, H. C., Wu, Y., Schulte, F., Friesen, M., Guo, R., et al. (2024). MECP2 directly interacts with RNA polymerase II to modulate transcription in human neurons. Neuron *112*, 1943–1958.e10. https://doi.org/10.1016/j.neuron.2024.04.007.

Loi, M., Trazzi, S., Fuchs, C., Galvani, G., Medici, G., Gennaccaro, L., Tassinari, M., and Ciani, E. (2020). Increased DNA Damage and Apoptosis in CDKL5-Deficient Neurons. Mol. Neurobiol. *57*, 2244–2262. https://doi.org/10.1007/s12035-020-01884-8.

Maranon, D.G., Sharma, N., Huang, Y., Selemenakis, P., Wang, M., Altina, N., Zhao, W., and Wiese, C. (2020). NUCKS1 promotes RAD54 activity in homologous recombination DNA repair. J. Cell Biol. *219*, e201911049. https://doi.org/10.1083/jcb.201911049.

Maxwell, S.S., Pelka, G.J., Tam, P.P., and El-Osta, A. (2013). Chromatin context and ncRNA highlight targets of MeCP2 in brain. RNA Biol. *10*, 1741–1757. https://doi.org/10.4161/rna.26921.

Nan, X., Cross, S., and Bird, A. (1998). Gene silencing by methyl-CpG-binding proteins. Novartis Found. Symp. *214*, 6–50. discussion 16–21, 46–50.

Negraes, P.D., Trujillo, C.A., Yu, N.K., Wu, W., Yao, H., Liang, N., Lautz, J.D., Kwok, E., McClatchy, D., Diedrich, J., et al. (2021). Altered network and rescue of human neurons derived from individuals with early-onset genetic epilepsy. Mol. Psychiatry *26*, 7047–7068. https://doi.org/10.1038/s41380-021-01104-2.

Ohashi, M., Korsakova, E., Allen, D., Lee, P., Fu, K., Vargas, B.S., Cinkornpumin, J., Salas, C., Park, J.C., Germanguz, I., et al. (2018). Loss of MECP2 Leads to Activation of P53 and Neuronal Senescence. Stem Cell Rep. *10*, 1453–1463. https://doi.org/10.1016/j.stemcr.2018.04.001.

Okumura, H., Hayashi, R., Unami, D., Isono, M., Yamauchi, M., Otsuka, K., Kato, Y., Oike, T., Uchihara, Y., and Shibata, A. (2025). MeCP2 deficiency leads to the γH2AX nano foci expansion after ionizing radiation. DNA Repair (Amst) *145*, 103790. https://doi.org/10.1016/j.dnarep.2024.103790.

Parplys, A.C., Zhao, W., Sharma, N., Groesser, T., Liang, F., Maranon, D.G., Leung, S.G., Grundt, K., Dray, E., Idate, R., et al. (2015). NUCKS1 is a novel RAD51AP1 paralog important for homologous recombination and genome stability. Nucleic Acids Res. *43*, 9817–9834. https://doi.org/10.1093/nar/gkv859.

Pecorelli, A., Leoni, G., Cervellati, F., Canali, R., Signorini, C., Leoncini, S., Cortelazzo, A., De Felice, C., Ciccoli, L., Hayek, J., and Valacchi, G. (2013). Genes related to mitochondrial functions, protein degradation, and chromatin folding are differentially expressed in lymphomonocytes of Rett syndrome patients. Mediators Inflamm. *2013*, 137629. https://doi.org/10.1155/2013/137629.

Penning, T.D. (2010). Small-molecule PARP modulators–current status and future therapeutic potential. Curr. Opin. Drug Discov. Devel. *13*, 577–586.

Prigione, A., Fauler, B., Lurz, R., Lehrach, H., and Adjaye, J. (2010). The senescence-related mitochondrial/oxidative stress pathway is repressed in human induced pluripotent stem cells. Stem Cell. *28*, 721–733. https://doi.org/10.1002/stem.404.

Quaderi, N.A., Meehan, R.R., Tate, P.H., Cross, S.H., Bird, A.P., Chatterjee, A., Herman, G.E., and Brown, S.D. (1994). Genetic and physical mapping of a gene encoding a methyl CpG binding protein, Mecp2, to the mouse X chromosome. Genomics *22*, 648–651. https://doi.org/10.1006/geno.1994.1442.

Rohani, L., Johnson, A.A., Arnold, A., and Stolzing, A. (2014). The aging signature: a hallmark of induced pluripotent stem cells? Aging Cell *13*, 2–7. https://doi.org/10.1111/acel.12182.

Samarasinghe, R.A., Miranda, O.A., Buth, J.E., Mitchell, S., Ferando, I., Watanabe, M., Allison, T.F., Kurdian, A., Fotion, N.N., Gandal, M.J., et al. (2021). Identification of neural oscillations and epileptiform changes in human brain organoids. Nat. Neurosci. *24*, 1488–1500. https://doi.org/10.1038/s41593-021-00906-5.

Sanchez, A., Buck-Koehntop, B.A., and Miller, K.M. (2022). Joining the PARty: PARP Regulation of KDM5A during DNA Repair (and Transcription?). Bioessays *44*, e2200015. https://doi.org/10.1002/bies.202200015.

Sikora, E. (2013). Rejuvenation of senescent cells-the road to postponing human aging and age-related disease? Exp. Gerontol. *48*, 661–666. https://doi.org/10.1016/j.exger.2012.09.008.

Singhal, N., Graumann, J., Wu, G., Araúzo-Bravo, M.J., Han, D.W., Greber, B., Gentile, L., Mann, M., and Schöler, H.R. (2010). Chromatin-Remodeling Components of the BAF Complex Facilitate Reprogramming. Cell *141*, 943–955. https://doi.org/10.1016/j.cell.2010.04.037.

Squillaro, T., Alessio, N., Capasso, S., Di Bernardo, G., Melone, M.A. B., Peluso, G., and Galderisi, U. (2019). Senescence Phenomena and Metabolic Alteration in Mesenchymal Stromal Cells from a Mouse Model of Rett Syndrome. Int. J. Mol. Sci. *20*, 2508. https://doi.org/10.3390/ijms20102508.

Squillaro, T., Alessio, N., Cipollaro, M., Renieri, A., Giordano, A., and Galderisi, U. (2010). Partial silencing of methyl cytosine protein binding 2 (MECP2) in mesenchymal stem cells induces senescence with an increase in damaged DNA. FASEB J. *24*, 1593–1603. https://doi.org/10.1096/fj.09-143057.

Squillaro, T., Hayek, G., Farina, E., Cipollaro, M., Renieri, A., and Galderisi, U. (2008). A case report: bone marrow mesenchymal stem cells from a Rett syndrome patient are prone to senescence and show a lower degree of apoptosis. J. Cell. Biochem. *103*, 1877–1885. https://doi.org/10.1002/jcb.21582.

Tanaka, Y., Kim, K.Y., Zhong, M., Pan, X., Weissman, S.M., and Park, I.H. (2014). Transcriptional regulation in pluripotent stem cells by methyl CpG-binding protein 2 (MeCP2). Hum. Mol. Genet. *23*, 1045–1055. https://doi.org/10.1093/hmg/ddt500.

Tropea, D., Giacometti, E., Wilson, N.R., Beard, C., McCurry, C., Fu, D.D., Flannery, R., Jaenisch, R., and Sur, M. (2009). Partial reversal of Rett Syndrome-like symptoms in MeCP2 mutant mice. Proc. Natl. Acad. Sci. USA *106*, 2029–2034. https://doi.org/10.1073/pnas.0812394106.

Vacca, M., Tripathi, K.P., Speranza, L., Aiese Cigliano, R., Scalabrì, F., Marracino, F., Madonna, M., Sanseverino, W., Perrone-Capano, C., Guarracino, M.R., and D'Esposito, M. (2016). Effects of Mecp2 loss of function in embryonic cortical neurons: a bioinformatics strategy to sort out non-neuronal cells variability from transcriptome profiling. BMC Bioinf. *17*, 14. https://doi.org/10.1186/s12859-015-0859-7.

Zhou, Z., Hong, E.J., Cohen, S., Zhao, W.N., Ho, H.Y.H., Schmidt, L., Chen, W.G., Lin, Y., Savner, E., Griffith, E.C., et al. (2006). Brain-specific phosphorylation of MeCP2 regulates activity-dependent Bdnf transcription, dendritic growth, and spine maturation. Neuron *52*, 255–269. https://doi.org/10.1016/j.neuron.2006.09.037.

Stem Cell Reports, Volume *20*

Supplemental Information

Probing DNA damage in Rett syndrome neurons uncovers a role for MECP2 regulation of PARP1

A. Morales, E. Korsakova, N. Mansooralavi, A. Ravikumar, G. Rivas, P. Soliman, L. Rodriguez, T. McDaniel, A. Lund, B. Cooper, A. Bhaduri, and W.E. Lowry

Supplementary Figures

Supplemental Figure 1. Supplemental images and flow cytometry plots for mitochondrial assays in Rett syndrome neurons. (A) Mitochondrial morphology in neurons was studied using confocal microscopy and immunostaining for Tom20. Images taken in 63X. (B) Flow cytometry plots of JC-1 treated Rett neurons to understand mitochondrial permeability. Data are quantified in Fig 2I. CCCP, a proton gradient uncoupler, was used as a positive control.

Figure S1

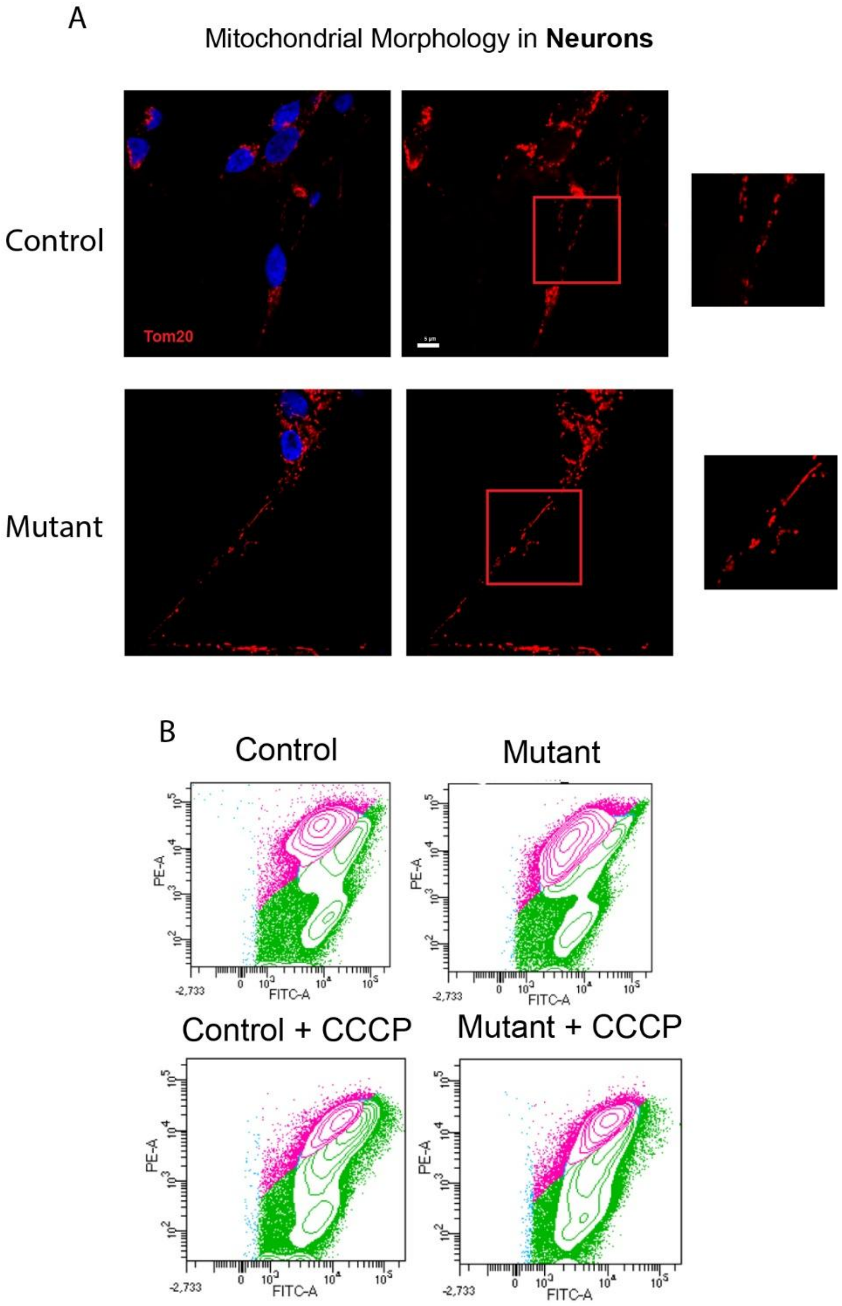

Supplemental Figure 2. Further analysis of published MECP2 protein interactomes. (A) Venn diagram of proteins identified from IP-mass spectrometry in wildtype and MECP2 mutant neurons (R15). (B) MECP2 protein interactome from OpenCell proteome database. (C) Gene ontology analysis of MECP2 interactors and their interactomes. (C) Left: Heatmap of Jaenisch proteomic database of 45 shared proteins from Figure 4E including values from Rett syndrome mutation R133C. Right: PARP1 protein quantity from Jaenisch IP-MS pull down between control and mutant neurons.

Figure S2

A

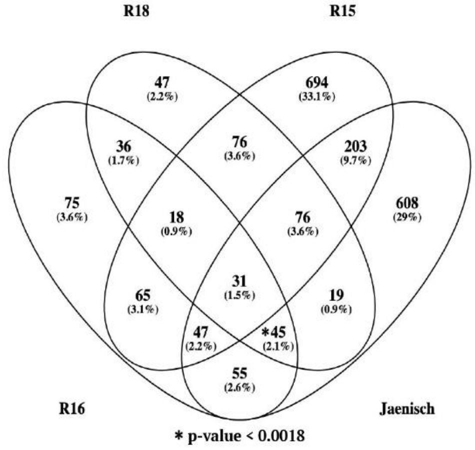

B

OpenCell MECP2 Interacations Table

Bait	Prey	P value (-log p)	Relative Enrichment	Cellular Abundance	Interaction stoichiometry	Cluster ID	Core complex
PARP1	MECP2	83.86	10.66	-2	-2.5	None	None
H2BC11	MECP2	68.6	6.55	-1.95	-2.76	None	None
MECP2	MECP2	39.64	8.33	0	0	302	336
MECP2	BAZ1A	25.43	6.14	0.41	-3.04	302	336
KPNA4	MECP2	12.32	4.15	-1.19	-2.54	None	None
MECP2	CHRAC1	9.93	4.7	0.43	-2.4	302	336
SNX1	MECP2	7.78	3.38	-0.71	-3.87	None	None
MECP2	MAPK1/4	6.85	3.19	1.66	0.01	None	None
NUCKS1	MECP2	2.56	2.06	-1.01	-2.54	None	None

Term	P value	Adjusted p value	Odds Radtio	Combined score
Chromatin Organization	6.32E-60	8.60495E-57	28.16207489	3838.820915
Chromatin Remodeling	1.53E-48	1.04273E-45	25.78368506	2838.733324
DNA Metabolic Process	3.82E-39	1.73507E-36	17.59838166	1556.754875
Regulation Of DNA-templated Transcription	7.51E-33	2.55781E-30	5.492278513	406.2573335
DNA Repair	2.2E-29	5.98668E-27	13.59036596	896.7947049
DNA Damage Response	1.18E-27	2.68858E-25	10.81257606	670.385825
Regulation Of Transcription By RNA Polymerase II	7.29E-23	1.41853E-20	4.217323749	214.9691231
Positive Regulation Of DNA-templated Transcription	1.4E-22	2.38605E-20	5.056130586	254.4211089
Negative Regulation Of Nucleic Acid-Templated Transcription	6.07E-20	9.18807E-18	7.61894788	337.1240452
Positive Regulation Of Double-Strand Break Repair	7.95E-17	5.69548E-15	20.78012685	770.3478647

C

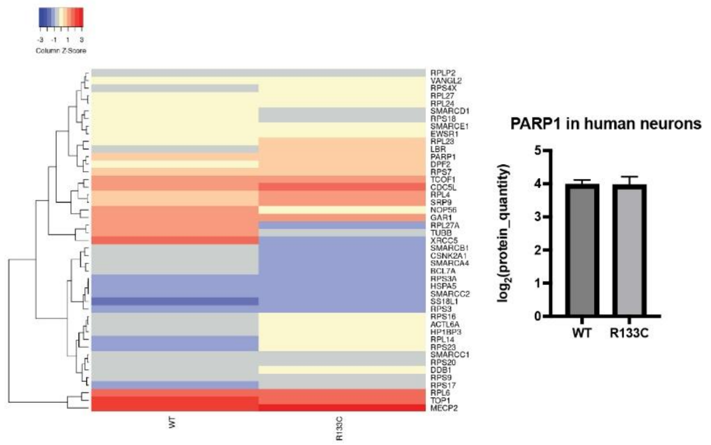

Supplemental Methods

Animals Generation of Rett syndrome rat model consisted of crosses of Mecp2ZFN/+ females (SD- Mecp2tm1sage) to wildtype (WT) S100b eGFP to produce Mecp2ZFN/y and Mecp2ZFN/+ rats. Details about this model are found in Patterson et al [32] and Morales et al (in press).

Library Preparation and Sequencing Isolated RNA (bulk RNAseq) were delivered to the UCLA Technology Center for Genomics & Bioinformatics where libraries for RNA sequencing were prepared. The samples were sequenced using NovaSeq 6000 S2 PE 2x50 with 50,000 reads per cell.

Disruption of mitochondrial function Cells were washed with PBS and treated with either DMSO, 1 uM Phenformin, 20 uM Phenformin, 0.1 uM Rotenone or 5 uM Rotenone for three days. Media was changed every day.

JC-1 Mitochondrial Permeability Assay Mitochondrial permeability was measured using ThermoFisher' s MitoProbe™ JC-1 Assay Kit (M34152) according to the manufacturer's protocol. A measure of permeability was calculated by a ratio of green and red flow cytometry events.

Reactive Oxygen Species Measurement Detection of ROS in live cells was conducted using ThermoFisher MitoSOX™ Mitochondrial Superoxide Indicators for live-cell imaging (M36007) according to manufacturer's directions.

RNA Expression Profiling RNA-seq was performed as described previously [61]. Data from experiments in figure 6 are available at Mendeley (Loss of MECP2 in human neurons - Mendeley Data)

Gene Ontology and Transcription Factor Analysis Analysis was performed using Enrichr, a gene list enrichment analysis tool. https://maayanlab.cloud/Enrichr/

Metabolic Pathway Analysis Analysis was performed using pathway analysis module of the MetaboAnalyst platform. https://www.metaboanalyst.ca/

Mitochondrial Morphology Analysis Mitochondrial morphology parameters were quantified using the MitochondrialAnalyzer plugin in ImageJ. https://github.com/AhsenChaudhry/Mitochondria-Analyzer

Confocal Colocalization Analysis The cells were analyzed by confocal microscopy using inverted confocal laser microscope Zeiss LSM880 with Airyscan at 100X. Pearson's Correlation Coefficient and was quantified using Zeiss's ZEN Blue's colocalization module.

Dendritic Branching Analysis The stained cells were then imaged at 20×, and dendritic arbors of individual cells were traced using ImageJ. The number of dendritic ends per cell were counted using the Cell Counter plugin for ImageJ. The number of dendritic ends per cell are presented as mean ends per cell ± SEM.

Western Blot Cell lysate was prepared using RIPA buffer (Pierce) supplemented with Halt Protease Inhibitor Cocktail (ThermoFisher Scientific) and Halt Phosphatase Inhibitor Cocktail (ThermoFisher Scientific). Total protein concentration was determined using BCA Protein Assay Kit (ThermoFisher Scientific) following the manufacturer's protocol. Equal protein concentrations were loaded onto the NuPAGE 4-12% Bis-Tris gel (ThermoFisher Scientific) and run at 150 Volts for 90 minutes in running buffer, containing 25 mL of 20x NuPAGE MOPS SDS Running Buffer (ThermoFisher Scientific)

and 475 mL of mili-Q water. Next, the protein was transferred onto the nitrocellulose membrane at 30 Volts for 60 minutes in transfer buffer, containing 25 mL 20x NuPAGE Transfer Buffer (ThermoFisher Scientific), 100 mL Methanol (ThermoFisher Scientific), 375 mL mili-Q water. The membrane was blocked overnight at 4°C in OneBlock Western-FL Blocking Buffer (Genesee Scientific), then incubated in the primary antibody at 4°C overnight. The following primary antibodies were used: rabbit VDAC (Cell Signaling Technology #4661, 1:1000), mouse β-actin (SCBT sc-47778, 1:500). The membrane was washed twice with 0.1% PBST and incubated in anti-rabbit or anti-mouse secondary HRP-labeled secondary antibody (ThermoFisher Scientific 31460, 31430 1:100000) for 1 hour at room temperature. The membrane was washed twice with 0.1% PBST and SuperSignal West Femto Maximum Sensitivity Substrate (ThermoFisher Scientific) was added to the membrane and subjected to film exposure.

C13 labeled glucose incorporation Cells were fed with DMEM (ThermoFisher Scientific) supplemented with 4 mM glutamine (ThermoFisher Scientific), 1 mM pyruvate (ThermoFisher Scientific), and 10 mM C13 labeled glucose (Cambridge Isotope Laboratories). 24 hours later the cells were washed twice with ammonium acetate (ThermoFisher Scientific) on ice and 80% methanol (ThermoFisher Scientific) was added to the cells. The plates were placed in -80°C for 15 minutes. Next, the cells were scraped off the plate into Eppendorf tubes, vortexed and centrifuged at 17000 g for 10 minutes at 4°C. The methanol was then evaporated using the EZ-Lite evaporator. Dried samples were analyzed using mass spectrometry with cell count normalization between samples.

Seahorse Assay Cell were plated at a density 50,000-90,000 cells per well in a XF96 microplate (Agilent) and placed in the 37oC 5% CO2 incubator overnight. The next day, the cells were washed twice with the assay medium (Dulbecco's Modified Eagle's Medium supplemented with 10 mM glucose, 2 mM L-glutamine, 1 mM pyruvate and 5 mM HEPES, pH 7.4), and the microplate was placed in a 37°C incubator without CO2. 30 minutes later, the plate was loaded into the Seahorse XF96 Extracellular Flux Analyzer (Agilent Technologies). The following compounds were Injected during the assay: 2 uM oligomycin, 0.75 and 1.35 uM FCCP; 2 uM rotenone and antimycin A. When the measurements were done, cells were fixed with 4% paraformaldehyde, stained with Hoechst, and cell number per well was determined using an Operetta High-Content Imaging System (PerkinElmer). Oxygen consumption rates (OCR) were normalized to cell number per well.

siRNA Gene Silencing MeCP2 protein knockdown was performed using Lipofectamine™ RNAiMAX Transfection Reagent (Invitrogen) kit. A reverse transfection method was used with a ratio of 1 μl lipofectamine:20 nM siRNA for each well of a 24-well plate. Briefly, Lipofectamine and oligos were premixed in 100 μl of OptiMEM (Gibco) for 20 min in the precoated receiving plate. Cells were then passaged with TrypLE (Gibco), resuspended in 500 μl of cell media without antibiotics, and plated on top of transfection media. Transfections were incubated overnight at 37°C after which time media was replaced with standard cell culturing media with antibiotics for the indicated lengths of time.

Cell Culture Treatments To reduce PARP activity, cells were treated with Olaparib (Selleck) at a concentration of 500 nM at indicated time points. To induce PARP activity,

β-Nicotinamide adenine dinucleotide hydrate (Sigma) was used at 10 mM at indicated time points. To induce DNA damage, cells were treated with etoposide (MP Biomedicals) at a concentration of 10 μM for 24 hours.

Generation of *in vitro* neurons First, isogenic Rett Syndrome and wild type human induced pluripotent stem cells (hiPSCs) were derived from Rett patient fibroblasts as described previously (Ohashi *et al.*, 2018). The CDKL5 lines were a generous gift from Allyson Muotri and are described elsewhere [29]. hiPSCs were maintained on plates coated with matrigel (Corning) in mTeSR1 (StemCell Technologies) until 80% confluency. Neural progenitor cell (NPC) fate was induced using StemDiff manufacturer's protocol. In our hands, this approach generates a uniform population of immature cortical neurons, as described previously (Patterson et al., 2012). EGF and FGF were removed from the media, and the cells were cultured in DMEMF12 supplemented with N2 and B27 (Thermo Fisher).
